# Comparison between Timelines of Transcriptional Regulation in Mammals, Birds, and Teleost Fish Somitogenesis

**DOI:** 10.1371/journal.pone.0155802

**Published:** 2016-05-18

**Authors:** Bernard Fongang, Andrzej Kudlicki

**Affiliations:** Department of Biochemistry and Molecular Biology, Sealy Center for Molecular Medicine, Institute for Translational Sciences, University of Texas Medical Branch, 301 University Blvd, Galveston, Texas, USA; Oxford Brookes University, UNITED KINGDOM

## Abstract

Metameric segmentation of the vertebrate body is established during somitogenesis, when a cyclic spatial pattern of gene expression is created within the mesoderm of the developing embryo. The process involves transcriptional regulation of genes associated with the Wnt, Notch, and Fgf signaling pathways, each gene is expressed at a specific time during the somite cycle. Comparative genomics, including analysis of expression timelines may reveal the underlying regulatory modules and their causal relations, explaining the nature and origin of the segmentation mechanism. Using a deconvolution approach, we computationally reconstruct and compare the precise timelines of expression during somitogenesis in chicken and zebrafish. The result constitutes a resource that may be used for inferring possible causal relations between genes and subsequent pathways. While the sets of regulated genes and expression profiles vary between different species, notable similarities exist between the temporal organization of the pathways involved in the somite clock in chick and mouse, with certain aspects (as the phase of expression of Notch genes) conserved also in the zebrafish. The regulated genes have sequence motifs that are conserved in mouse and chicken but not zebrafish. Promoter sequence analysis suggests involvement of several transcription factors that may bind these regulatory elements, including *E2F*, *EGR* and *PLAG*, as well as a possible role of G-quadruplex DNA structure in regulation of the cyclic genes. Our research lays the groundwork for further studies that will probe the evolution of the regulatory mechanism of segmentation across all vertebrates.

## Introduction

Metameric segments of the vertebrate body are derived from somites, distinct fragments of paraxial mesoderm. Somitogenesis (somite formation) is controlled by a periodic molecular oscillator termed the segmentation clock. The process, which is precisely regulated in both space and time, depends on waves of gene expression progressing through the presomitic mesoderm (PSM) along the antero-posterior (AP) axis of the body [1–3], and involves transcriptional regulation of genes, mainly from the Notch, Fgf and Wnt signaling pathways [4, 5]. Expression of each regulated gene is confined to its respective time interval. In the mouse genes from each pathway are transcribed in a specific phase, also the order of transcriptional activation of genes within each phase is intricately regulated. The length of the somite cycle, and also the number of somites varies across species: 30 minutes per cycle (over approx. 30 cycles) for zebrafish, 90 minutes (over approx. 50 cycles) for chick, and 120 minutes (over approx. 65 cycles) for mouse. The waves of gene expression interact with morphogen gradients to set the time in cells at which they will form a somite. This “clock and wavefront” model agrees with observations in the chick, the zebrafish [6–8] and mouse [1, 9]. To date, significant number of genes has been reported as cyclic during the process and they primarily belong to the Wnt, Fgf or Notch signaling pathway. In a recent study by Dias et al, somite-like bodies were formed also without a global gradient [10]; it is possible that transcriptional oscillations may be inherent to every cell in the PSM, while the rostrocaudal waves and morphogen interactions are responsible for synchronizing the process.

Notch signaling is a crucial element of the master somite oscillator since most of the well characterized cyclic genes are members of this pathway. It has been reported that in absence of Notch signaling, no oscillations are observed and somites form [9]. In the mouse, chicken, and zebrafish, known Notch cyclic genes include: *Hes1/7/5*, *her1/2/47/15*, *deltaC*, *Nup37*, *Hey1/3*, *Lfng*, *Nkd1*, *Nrarp*, *Maml3*, *Bcl9l* [11]. The Wnt signaling pathway is also rhythmically activated in the PSM and reported cyclic genes from this pathway include *Axin2*, *Dact1*, *Dkk1*, *Sp5*, *Tnfrsf19*, *Myc*, *Has2*, *T*, and *Phlda1*. Also, several studies have confirmed that inactivation of Wnt cyclic genes such as *Dkk1* creates segmentation defects in several vertebrates [8, 11–14]. The Fgf signaling pathway has also been shown to be rhythmically regulated during somite development, where genes like *Spry2/4*, *Dusp6*, *Shp2*, *Hspg2*, *Efna2*, and *Bcl2l11* display oscillatory expression patterns. In a cross-comparison study of cyclic genes in the mouse, zebrafish and chick, Krol et al [8] found that only *Hes1* and *Hes5* orthologs showed cyclic expression in all three species.

Transcriptional regulation during somitogenesis has been studied using microarrays of PSM tissue [4, 8]. In these experiments, samples were collected from embryos, to cover one or more somite cycles, and transcripts were hybridized to probes on microarray chips. We recently developed and implemented a method [15], based on spatiotemporal maximum entropy deconvolution, that assigns the correct phase of the cycle to each data point, characterizes the dependence between time, position and cycle phase, performs the deconvolution to reconstruct the full spatiotemporal profile, determines the phase of expression peak, and estimates the accuracy and resolution of the resulting timing of each gene involved in the somite formation process. The reconstruction of the spatiotemporal profile allowed inferring the network of causal relations in mouse somitogenesis. We established the hierarchy between the signaling pathways: Wnt signaling acts downstream of Notch, which in turn acts downstream of Fgf. We also identified genes with two peaks of expression during a somite cycle. Our method was originally tailored to analyzing the experiments of Pourquie et al [4]; here we adapt it to other somitogenesis gene expression profiling experiments by adjusting the kernel function of the deconvolution.

In eukaryotes, the information controlling gene transcription is largely contained in the promoter region of the gene, usually defined as the sequence of 200 to tens of thousands nucleotides upstream of the Transcription Start Site (TSS). Since regulatory motifs are arranged in specific configurations that confer upon each gene an individualized spatial and temporal transcription program, it is believed that genes exhibiting similar expression patterns would share similar regulatory elements in their promoters. Therefore, finding transcription factor binding sites (TFBSs) for co-regulated genes may help elucidate the general mechanism that regulates these genes [16–18]. Genes regulated during somitogenesis are classified according to their signaling pathway affiliation and genes from the same pathway often share a similar expression profile [15]. One approach to investigate how oscillatory gene expression is produced in somitogenesis and to understand the crosstalk between pathways is to identify transcription factor binding sites or other regulatory motifs overrepresented in their promoters.

We have previously inferred the precise timeline of expression during the mouse somite cycle. The primary objective of this study is to obtain such high-resolution timelines also for zebrafish and chick, which will allow comparing the temporal structures of the transcriptional programs in mammals, birds, and teleost fish somitogenesis. To this end, we extend the Maximum Entropy deconvolution method previously established to two other vertebrates: the zebrafish and chicken. As the study of both species is based on the same principle as in the mouse [4, 8], we used the previously established suite of algorithms to reconstruct the spatiotemporal expression profiles in zebrafish and chicken somitogenesis. The inferred timelines show that the hierarchy between pathways observed in the mouse is generally conserved in the chicken. Also, the timelines of gene expression established in this study constitute a valuable resource that can be used to assess causation from time sequence. Finally, using a *de novo* motif finding approach, we identified regulatory motifs in the promoter of mouse cyclic genes and determined putative TFs that may bind to these sites. As most promoters of mouse cyclic genes are GC rich, we also investigated overrepresentation of DNA sequences potentially forming G-quadruplex DNA structures [19, 20] and their potential role in the regulation of oscillatory gene expression.

## Results and Discussion

### 2.1. Timing of gene expression during chicken and zebrafish somitogenesis

We applied the spatiotemporal deconvolution algorithm, which recover original signals from observed time course data, to the data of Krol et al [8] with the aim of discovering additional cyclic genes and inferring accurate timing for regulated genes. These published data are genome-wide mRNA concentrations in the tails of 18 and 21 embryos at different stages of the somite cycle of chicken and zebrafish, respectively. mRNA concentrations from the right posterior half PSM of the embryos where obtained using microarray while the rest of the embryo was used for retrospective positioning along the clock cycle as described in [4]. The results are related to the mouse spatiotemporal profiles and regulated genes described in [15]; all analyses assume a 120min, 30min and 90min period length for mouse, zebrafish and chicken, respectively.

As in the mouse [15], we used the spatiotemporal deconvolution algorithm (see Methods) to extract individual profiles for each gene involved in chicken and zebrafish somitogenesis, and the time of expression peak. In the mouse, genes related to the Wnt pathways peak early in the process (0- 45min of the 2 hour cycle); while Notch and Fgf related genes follow later. One important task during this work was to check whether the hierarchy between the three pathways is conserved in the chicken and zebrafish.

In the chick, we detected oscillating genes using the Lomb-Scargle (LS) test [21] (Methods) resulting in 263 probesets (95% confidence). We used deconvolution to reconstruct the original profiles of these genes, and to estimate the time of expression peak as well as its resolution. We selected 211 high-confidence probe sets with regular profiles and timing error less than 10 minutes. This list contains most of the previously reported cyclic genes including *HES1 (~72 min*: activated approximately 72 minutes after the beginning of the somite cycle), *HES5 (~78min)*, *T (~49min)*, *AXIN2 (~31min)*, *ID1 (~67min)*, *HAS2 (~40min)*, *IPO5 (~78min)*, and *LFNG (~78min)*, but also new candidate cyclic genes including *DOCK7 (~87min)*, *GRB2 (~51min)*, *AZI2 (~77min)*, *PLOCG2 (~75min)*, *RBM45 (~22min)*, and *NAALADL2 (~75min)*–previously not reported as cyclic during chicken somitogenesis. The beginning of the cycle is defined as the time at which the first band of *LFNG* is observed in the PSM. Table 1 shows the top 20 genes with one peak of expression and the full list is provided in S1 Table.

**Table 1:** Top 20 genes with one peak of expression during chicken somitogenesis. Times in minutes, assuming a 90mn periodicity, are provided for each peak. Accuracy has been estimated using a Monte Carlo method. All experimentally validated cyclic genes were detected using our algorithm (see [15]).

Probe set ID	Gene	Time(min)	LS p-value
Gga.3180.1.S2_a_at	*LFNG*	78±5	0.00345
Gga.3754.2.S1_at	*HES1*	72±4	0.00442
Gga.11242.1.S1_at	*HES5*	78±6	0.00477
GgaAffx.23401.4.S1_s_at	*DOCK7*	87±6	0.00531
Gga.14954.1.S1_at	*NAALADL2*	75±8	0.00615
GgaAffx.7254.1.S1_at	*AZI2*	77±4	0.00704
GgaAffx.22378.1.S1_s_at	*PLCG2*	75±6	0.00712
Gga.3772.1.S1_a_at	*T*	49±2	0.00737
Gga.6311.1.S1_at	*HEY1*	70±3	0.02147
Gga.329.1.S1_at	*HAS2*	40±3	0.0198
Gga.13220.1.S1_at	*DUSP22*	9±3	0.02493
Gga.2701.1.S2_at	*FGF3*	76±7	0.02595
Gga.12366.1.S1_at	*NUP37*	75±5	0.02417
GgaAffx.23741.1.S1_at	*DACT1*	81±6	0.0218
GgaAffx.25111.1.S1_s_at	*IPO5*	78±1	0.02073
Gga.4283.1.S1_at	*CTNNB1*	51±3	0.02377
Gga.892.1.S1_at	*ID1*	67±1	0.03083
Gga.8363.1.S2_at	*AXIN2*	21±8	0.03148
Gga.8082.1.A1_at	*RBM45*	22±7	0.03127
Gga.2170.1.S1_at	*GRB2*	51±8	0.04218

*DOCK7 (dedicator of cytokinesis 7)*, also known as *ZIR2*, is a member of the DOCK family of guanine nucleotide exchange factors (GEFs) which function as activators of small G proteins. It has been shown to be active during embryogenesis and plays a role in axon development [22], but has never been reported to play any role during somitogenesis.

Previously, we reported the existence of genes with two peaks of expression during each cycle of mouse somitogenesis. We found 141 such transcripts in chicken (84 high confidence, see Methods) that include *BBS9 (~6 and 56min)*, *CEP55 (~30 and 76min)*, *RAF1 (~12 and 78min)*, *SUCLA2 (~8 and 53min)*, *MLEC (~13 and 56min)*, *MAP3K7 (~9 and 58min)*, *RAB33B (~35 and 83min)*, *ORAOV1 (~23 and 65min)*, *XAB1 (12 and 56min)*, *PDCD4 (~38 and 85min)*, and *ARPC2 (~14 and 60 min)*. Details containing the probe set ID, the timing, and estimated errors of the genes with two peaks of expression, are provided as supplementary material (S2 Table).

**Fig 1:**
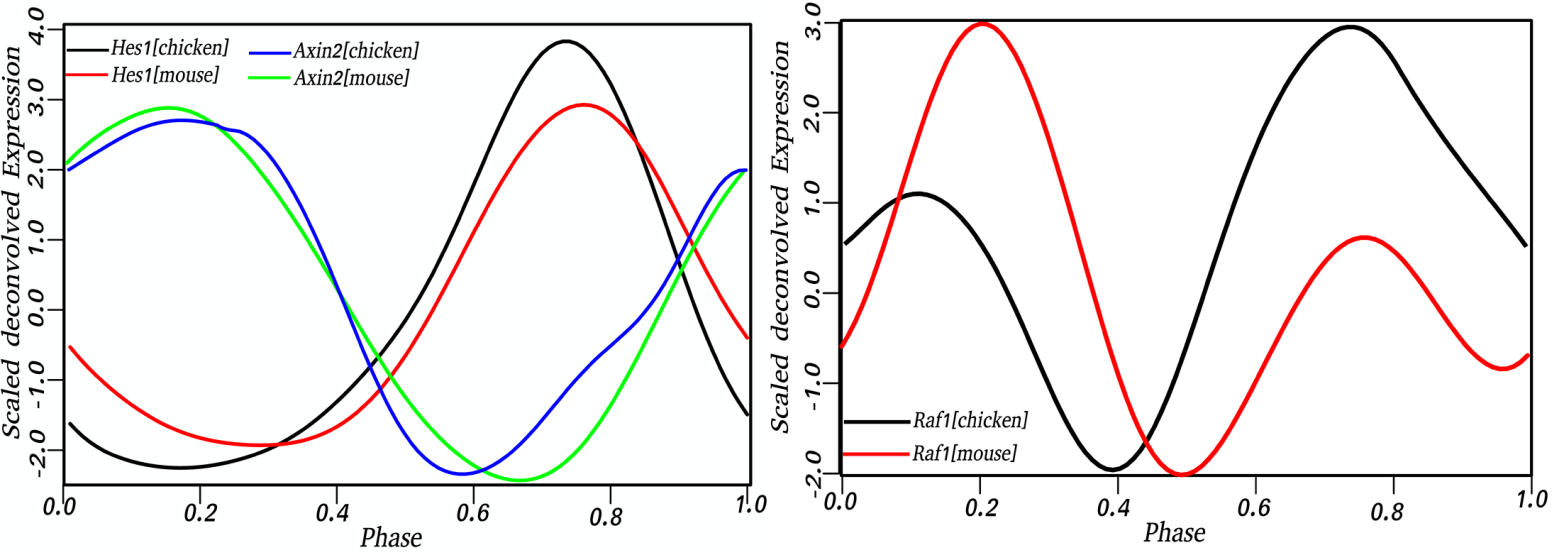
Phase conservation between mouse and chicken. *Axin2*, Wnt-related cyclic gene oscillates opposite phase to *Hes1* (Notch-related gene) suggesting a phase conservation between mouse and chicken. *Raf1*, an Fgf-related gene previously shown to have two peaks of expression during mouse somitogenesis showed the same pattern of expression in chicken. Phase of gene expression varies from 0 to 1 and corresponds to 120 and 90 minutes periodicity for mouse and chicken respectively.

We previously reported that *Raf1* (*v-raf-leukemia viral oncogene 1*), known to indirectly regulate members of the Fgf signaling pathway during mouse somite development, peaks twice during the mouse somite cycle [15]. We also suggested this double expression of *Raf1* may explain, in part, why some Fgf cyclic genes were opposite phase to others. The expression profile of *Raf1* during chicken somite development is very similar as it displays 2 peaks of expression (Fig 1). This similarity of expression profile suggests *Raf1* may be playing the same role as in the mouse by regulating the Fgf cyclic genes active in the opposite phase of the cycle.

Relating the timeline of somitogenesis in land vertebrates to zebrafish is an important step toward discovering the general properties of the mechanism in all vertebrates, as the zebrafish genome is an early branch compared to the common ancestor of birds and mammals. In the data of ([8]), we identified 111 transcripts with a regular oscillatory pattern showing one peak per cycle (Methods). Genes in this category include *dlc (~17min)*, *her4*.*2 (~25min)*, *her7 (~24min)*, *her1 (~16min)*, *her15*.*1 (~24min)*, and *her2 (~27min)* with established roles in zebrafish somite development, along with new candidates, as *rps29 (~14min)*, *spty2d1 (~2min)*, *utp11l (~28min)*, *mphosph8 (~13min)*, *prrx1a (~29min)*, *mrpl40 (~24min)*, *ech1 (~28min)*, and *nipa2 (~14min*.*)* (See Table 2 and S3 Table).

**Table 2:** Top 20 genes with one peak of expression during zebrafish somitogenesis. Times in minutes, assuming a 30mn periodicity, are provided for each peak. Accuracy has been estimated using a Monte Carlo method. All experimentally validated cyclic genes were detected using our algorithm (see [15]).

Probe set ID	Gene	Time(min)	LS p-value
Dr.8086.1.S1_s_at	*dlc*	17±1	0.0009
Dr.5372.1.S1_x_at	*her4*.*2*	25±1	0.0014
Dr.3696.1.S1_at	*her7*	24±1	0.0023
Dr.24815.2.S1_at	*rps29*	14±4	0.0024
Dr.11157.1.S1_at	*LOC100003640*	25±2	0.0039
Dr.1462.1.S1_at	*her1*	16±1	0.0044
Dr.5759.1.A1_at	*hoxd11a*	6±2	0.0053
Dr.1899.1.S1_at	*her15*.*1*	24±1	0.0058
Dr.4733.1.A1_at	*spty2d1*	2±1	0.0063
Dr.7852.1.S1_at	*utp11l*	28±4	0.0072
Dr.8835.2.S1_at	*mphosph8*	13±4	0.008
Dr.1410.1.S1_at	*prrx1a*	29±5	0.0083
Dr.4295.1.S1_at	*rfc4*	10±3	0.0087
Dr.17281.1.A1_at	*zgc*:*152990*	18±4	0.0097
Dr.1460.1.S1_at	*her2*	27±2	0.0106
Dr.12851.1.S1_at	*mrpl40*	24±9	0.0109
Dr.14998.2.S1_at	*ech1*	28±6	0.0111
Dr.14781.1.S1_at	*ubxn7*	14±3	0.012
Dr.17394.1.A1_at	*lmf2b*	18±7	0.0129
Dr.13969.1.A1_at	*nipa2*	14±3	0.0129

In addition, we identified 65 probe sets (32 regular) with two peaks of expression per cycle (see Methods and S4 Table), including *atn1 (~2 and 15 min)*, *ccdc22 (~7 and 22 min)*, *tfam (~10 and 25 min)*, *ppp2r5eb (~8 and 22 min)*, *arglu1b (~8 and 23 min)*, and *lzts2a (~2 and 15 min)*. None of these genes have been previously reported to have any role in zebrafish somitogenesis, however some like *atn1* have been shown to have function in brain development [23].

At the molecular level, the regulation of somite formation is still not fully understood and the determination of cyclic genes is just one step toward uncovering the mechanism regulating the process. As mentioned earlier, it is possible that genes with two peaks of expression may be as important as those with one peak, although their regulation is more difficult to study experimentally. Their presence in other species points toward conservation of the phenomena between vertebrates. Krol et al [8] have shown that the process of somite formation is not well conserved at the gene level but consistently involves the Wnt, Fgf and Notch signaling pathways. Indeed, in a comparative analysis, only 2 genes (*Hes1* and *Hes5*), both from the Notch pathway, were found to be cyclically expressed in all three species. It is likely that while the pathway and its modulation are essential, it may be sufficient that a subset of the genes is transcriptionally regulated, and the subsets are different in different species. Such conservation of a pathway or a process, albeit with a different subset of regulated genes, has been observed in other systems [24]. Our findings suggest that this may also apply to the groups of genes up-regulated twice per cycle that may function as a means of transcriptional coupling between the opposite phases.

### 2.2. Conservation of cyclic genes between mouse, chicken, and zebrafish somitogenesis

**Fig 2:**
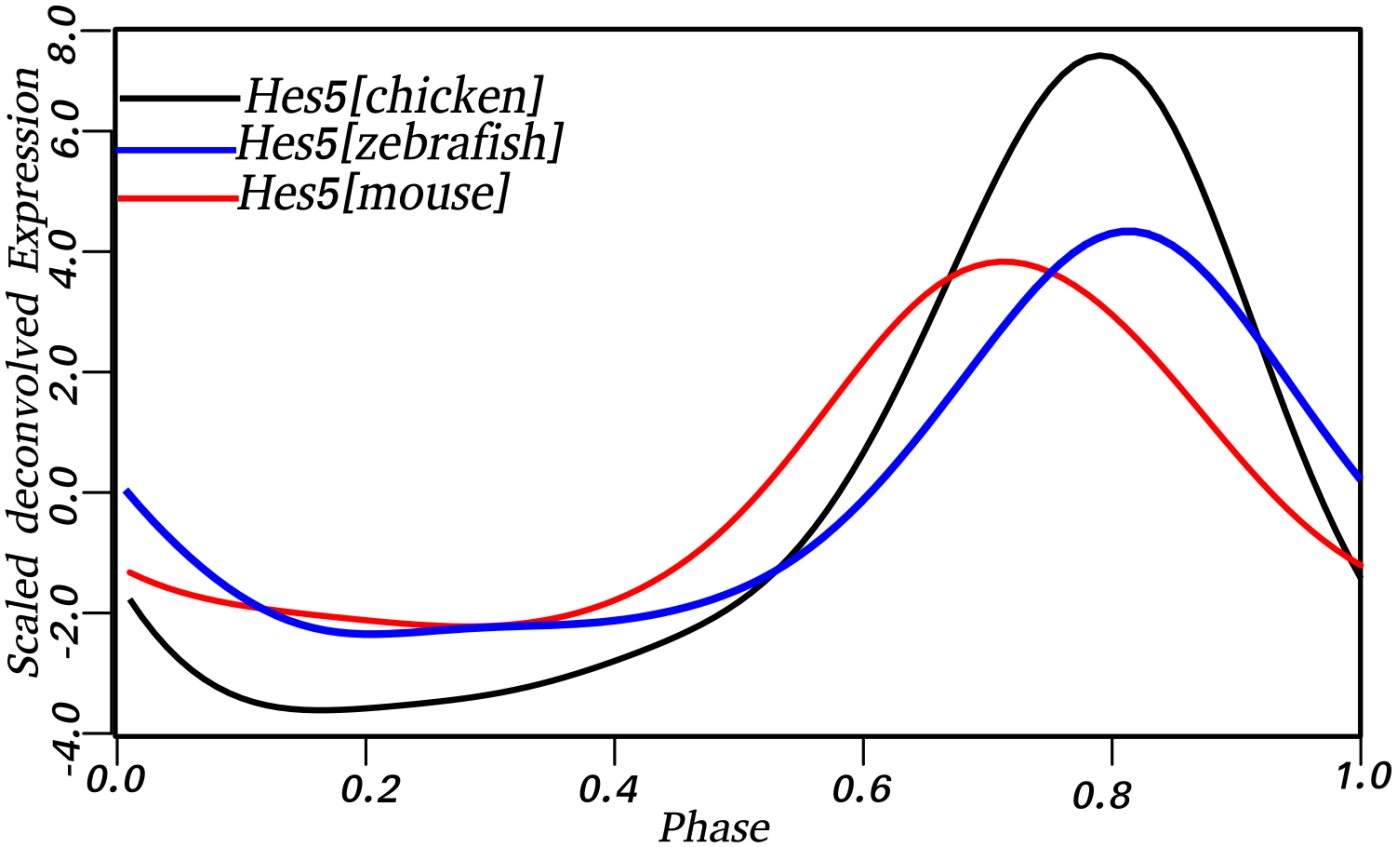
*Hes5* cyclic expression is conserved between mouse, chicken and zebrafish. The expression pattern of *Hes5* is shown as periodic during mouse, chicken and zebrafish somitogenesis. Moreover, the oscillations are in phase in all three species as depicted by the expression profile. Phase of gene expression varies from 0 to 1 and corresponds to 120, 90, and 30 minutes periodicity of mouse, chicken, and zebrafish respectively.

By precisely computing the timing of gene expression during mouse somitogenesis in a previous study, we proposed a list of 164 candidate cyclic genes with one peak, and a network of causality between these genes based on reported co-regulation. The present study led to 211 and 111 candidate cyclic genes with one peak of expression during chicken and zebrafish somitogenesis, respectively. A homology search (Methods) to find cyclic genes conserved between the three species confirms previously reported conclusions that somitogenesis is not well conserved at the gene level. Krol et al found in their cross-species analysis that only *Hes1* and *Hes5* were conserved between the three species. In this study, the precise timing of gene expression revealed only one gene, *Hes5* to be conserved between the species. The noisy expression profile of *her6*, the zebrafish homolog of *Hes1* (homology evidenced in [25]), did not allow timing of expression with an acceptable resolution (estimated timing error > 20 min). Additional candidate cyclic genes were conserved between the mouse and chicken (*Smc6*, *Urm1*, *Ube2l3*, *Bpgm*, *Wdr33*, *Msi2*), and *ATP5L* between the chicken and zebrafish. To our knowledge, these genes have not been previously reported as cyclic. The peak of expression of *Hes5* (*her15*.*1* for zebrafish), is in phase in all three species (Fig 2) suggesting it may play a critical role during the process.

The lack of conservation of the identities of the cyclic genes between species was also observed for bimodal genes. Indeed, no cyclic gene with two peaks of expression was found to be conserved between the mouse, chicken and zebrafish, and only one gene, *RAF1*, was found to be conserved between the mouse and chicken.

It should be noted that lack of evidence for conserved regulation does not constitute evidence of lack of regulation. For example in case of *ATP5L*, the gene was detected in mouse but we did not have sufficient evidence of conserved expression profile due to a low resolution (similar to *Hes1*). Additionally, zebrafish homologs of *Smc6*, *Urm1*, *Ube2l3* and *Msi2* were simply not present on the array while *Bpgm* and *Wdr33* were removed from analysis of zebrafish dataset for having too many affy-A calls, deeming the expression data unreliable. It is therefore possible that conserved profiles exist in additional genes, but could not be positively detected in the microarray studies for technical reasons.

### 2.3. Conservation of the hierarchy of pathways between mouse and chicken

The hierarchy between Wnt, Notch, and Fgf pathways has been a subject of debate for the past decade. The general consensus is that the Wnt pathway acts downstream of Notch, which in turn may be downstream of Fgf. We have previously shown that this global picture holds for the mouse somitogenesis [15]. The mouse somitogenesis clock is divided into two main parts representing genes oscillating in opposite phase. Wnt-related cyclic genes peak early whereas Notch and Fgf-related genes have their peak of expression later in the process. Moreover, although Notch and Fgf are nearly in phase, we have previously shown that the Notch pathway may be slightly downstream of Fgf.

**Fig 3:**
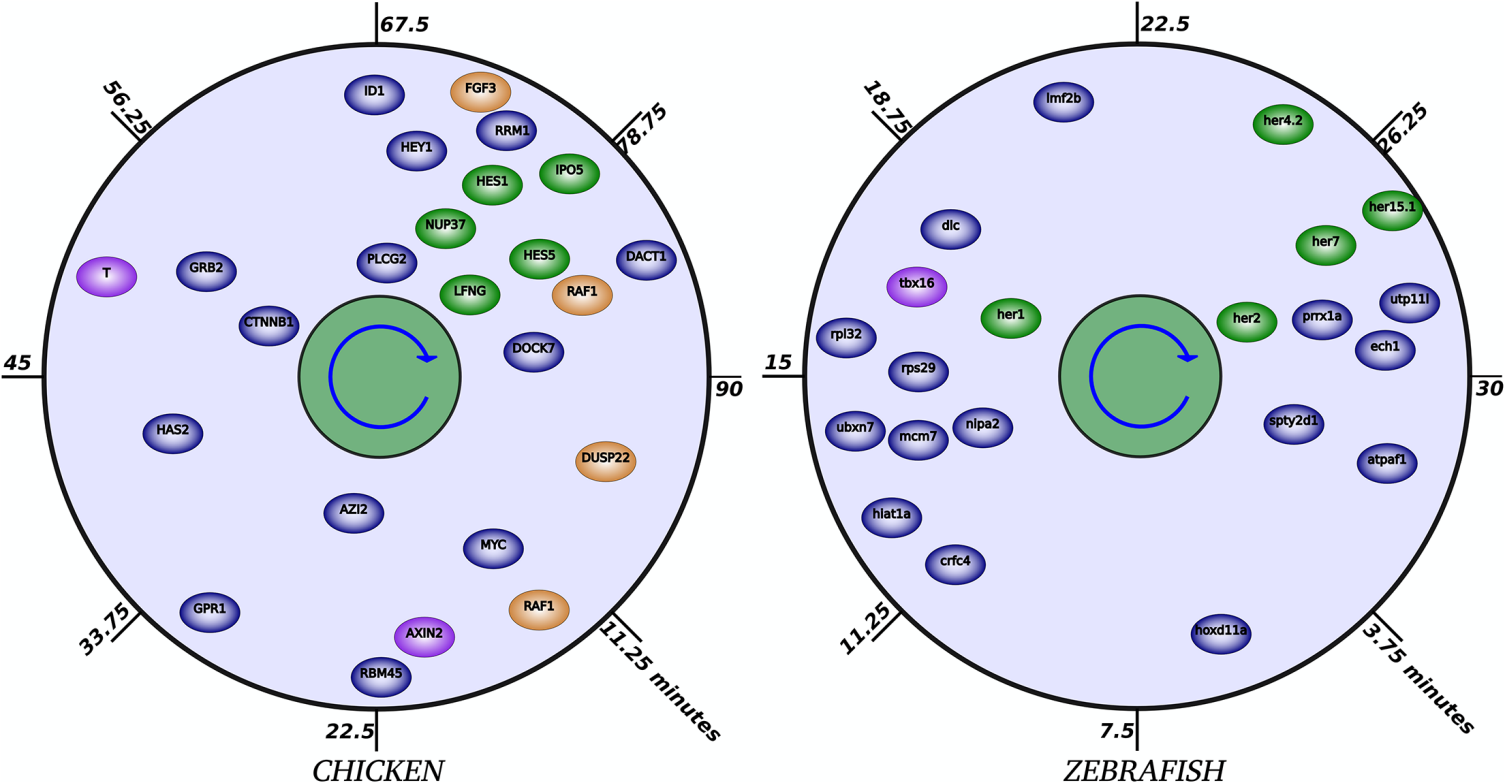
The timing of the significant genes involved in the chicken and zebrafish somitogenesis. Position of a gene symbol on the plots reflects time of peak timing (angle; clockwise) and the mean expression level (genes with high expression level are closer to the center). The timing is based on 90 and 30 minutes periodicity for the chicken and zebrafish somite cycle respectively. Genes are color-coded according to their known pathway association with green for Notch, brown for Fgf, and purple for Wnt. Genes in blue have never been reported as cyclic but are regulated during chicken and zebrafish somitogenesis. Also, a comparison with Fig 3 of [15] suggests that notable similarities exist between the temporal organization of the pathways involved in the somite clock in chick and mouse, with certain aspects (as the phase of expression of Notch genes) conserved also in the zebrafish.

Unlike zebrafish where the number of known cyclic genes is not sufficient to positively infer hierarchy between pathways, the timing of gene expression in chicken does suggest a relationship between the Wnt, Notch, and Fgf pathways. Indeed, as observed in Fig 3, two groups of genes oscillating in opposite phases are present in chicken. The first zone, from 11 to 56 minutes after the beginning of the cycle, contains Wnt-cyclic genes (*Axin2* and *T*) while the second group (with most genes clustered between 70 and 85 minutes of the cycle), contains Notch-cyclic genes (*LFNG*, *IPO5*, *HES1*, *HES5*, and *NUP37*) and Fgf-cyclic genes (*FGF3*, *DUSP22* and the first peak of *Raf1*). Although the regulatory clock does not contain all known cyclic genes, we expected that, as in the mouse, the remaining genes would fall into the proper category if the timing of expression is available. Fig 1 provides an example of the conservation of the profiles of key genes between the mouse and chicken. Wnt-cyclic gene *Axin2* oscillates in opposite phase of Notch-cyclic gene *Hes1* in both species, suggesting Wnt pathways may be acting downstream of Notch as in the mouse.

### 2.4. Functional annotation of the genes regulated in mouse, chicken, and zebrafish

**Fig 4:**
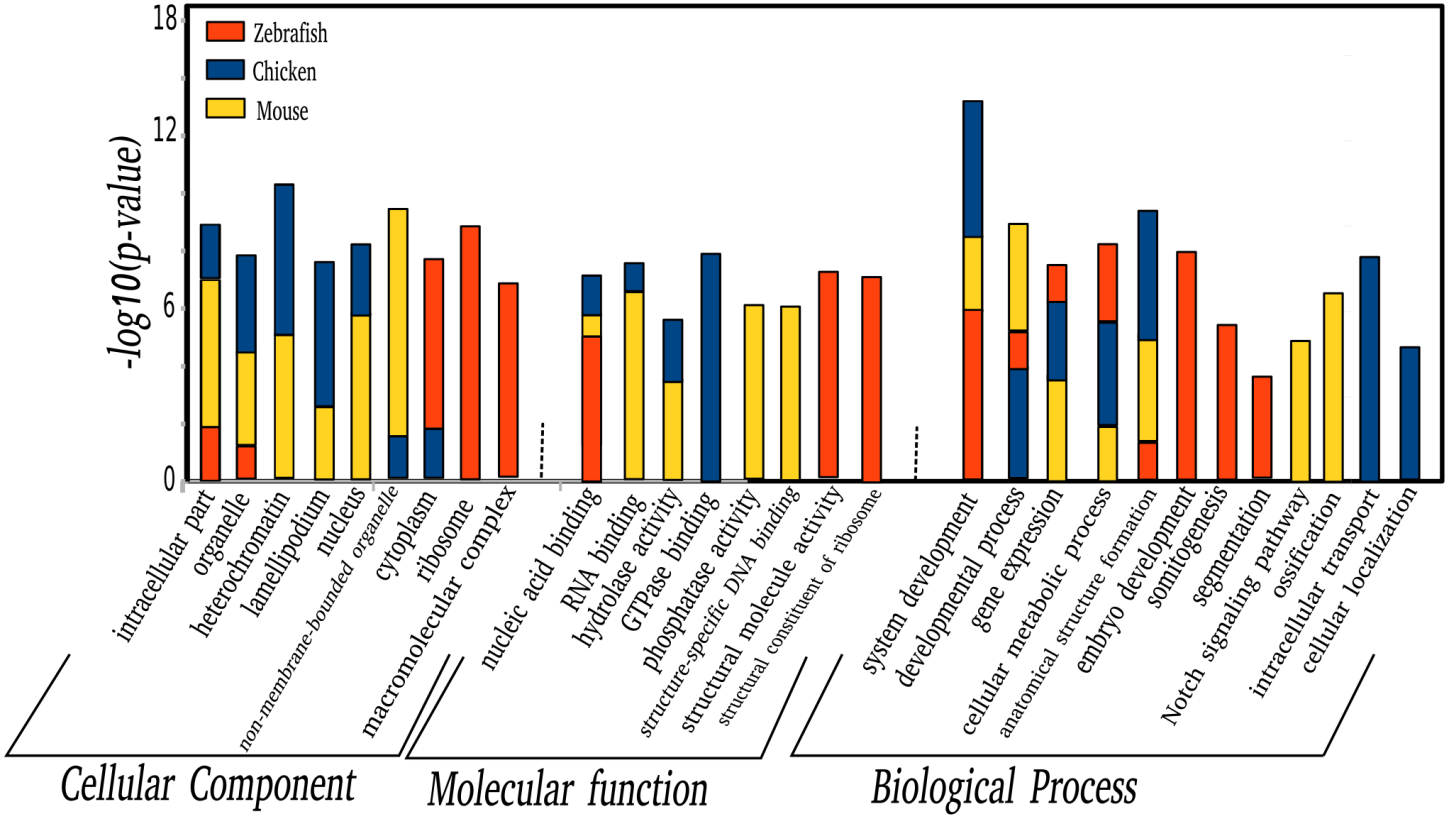
Gene Ontology analysis of the candidate cyclic genes with one peak of expression during mouse, chicken, and zebrafish somitogenesis. Shown are the top three GO terms for each species. The analysis was done using DAVID, an online set of tools for functional analysis of co-regulated genes. Only GO terms containing at least 5 of the input genes and a q-value (Benjamini corrected p-value) < 0.05 were selected.

To better understand the underlying regulations, we identified the conserved Gene Ontology (GO) terms overrepresented in the regulated genes (using the DAVID toolset [26]; see Methods), the results are shown in Fig 4. The genes cyclic in mouse, chicken, and zebrafish somitogenesis are linked to the same developmental processes; they also have a common molecular function (nucleic acid binding), consistent with a role in transcriptional regulation. Mouse and chicken candidate cyclic-genes share additional cellular components GO terms not enriched in zebrafish. This distribution of ontology terms associated with the regulated genes, combined with the fact that key cyclic genes like *Hes1* and *Axin2* shared the same expression profile, suggests a functional similarity at the gene level between mouse and chicken somitogenesis.

### 2.5. Transcriptional regulation of gene expression during mouse somitogenesis

While the global mechanism of somite formation is explained by the “clock and wavefront” model, regulation on the molecular level is yet to be deciphered. To address this question, we asked whether the cyclic genes may share common regulatory motifs, either specific or common to the main pathways involved in the process. We searched the upstream regions (promoters) of co-regulated genes (Wnt-, Notch-, and Fgf-cyclic genes) for binding sites of known TFs, as well as for *de novo* enriched regulatory sequences occurring at an unusual frequency. To this end, we compiled, from published data, experimentally verified cyclic genes for mouse, chicken and zebrafish somitogenesis and grouped them according to their signaling pathway (Table 3). Since the description of the somitogenesis process is more complete in the mouse than other species, cyclic genes from this species were used for motif enrichment analysis applying the MEME software suite [27] to the promoter DNA sequences. Significant motifs were subsequently identified in chick and fish promoter sequences (see Methods). We used 2000 bp upstream of TSS as a compromise between specificity and sensitivity of the search.

**Table 3:** Published cyclic genes of mouse, chicken and zebrafish somitogenesis used for motifs enrichment. We considered cyclic genes in the same pathway as co-regulated and used their sequences for cis-regulatory elements finding. Moreover, as somitogenesis is better described in mouse that any other species, mouse sequences are used for de novo motif finding and the subsequent motifs were used for enrichment analysis in chicken and zebrafish.

	Mouse	Chicken	Zebrafish
**Wnt**	*Axin2*[28], *Myc*[4], *Has2*[4], *Dkk1*[4], *Dact1*[29], *Foxo3a*[8], *Ccnd1*[8], *Ephb4*[8], *Lrig3*[8], *Sp5*[4], *Sp8*[8], *Tnfrsf19*[4], *Phlda1*[4]	*TRRAP*[8], *GARNL1*[8], *T* [8], *SRC* [8], *PSMF1*[8], *AXIN2*[8], *GPR177* [8], *RRM2*[8]	*tbx16*[8]
**Notch**	*Hes1*[30], *Lfng* [5], *Nrarp* [4], *Nkd1* [31], *Bcl9l*[4], *Hes5*[32], *Id1*[8], *Hes7* [33], *Huwe1*[8], *Skil*[8], *Hey1*[4], *Hey2*[34], *Id2*[8], *Ankhd1*[8]	*NUP93*[8], *NUP37*[8], *IPO5*[8], *NUP155*[8], *IPO11*[8], *HAIRY1*[30], *HAIRY2*[30], *HEY2*[34], *LFNG*[5], *SEPT7*[8], *GPS1*[8], *HES5*[8], *HES1*[9]	*her1*[35], *her7*[36], *her11*[8], *her12*[8], *her15*[8], *dlc*[37], *nrarp*[38]
**Fgf**	*Spry2*[4], *Efna1*[4], *Hspg2*[4], *Egr1*[4], *Shp2*[4], *Dusp1*[4], *Spry4*[39], *snail1*[4], *Dusp6* [4], *Hspg2*[4], *Bcl2l11*[4], *Dusp4*[40]	*RAF1*[8], *FGF3*[8], *SNAIL2*[41], *DUSP2*[8], *SDC2*[8], *MAP2k2*[8], *DUSP22*[8], *SNAIL1*[8], *DUSP6*[8]	*tbx16*[8], *rhov* [8]

While the enrichment of the identified motifs is statistically significant, their presence may be related to a cofounding factor and therefore an experimental study will be required to confirm the role of these elements in transcriptional regulation during the somite cycle.

### 2.6. Wnt, Notch, and Fgf-cyclic genes share common DNA motifs in their promoter regions

**Fig 5:**
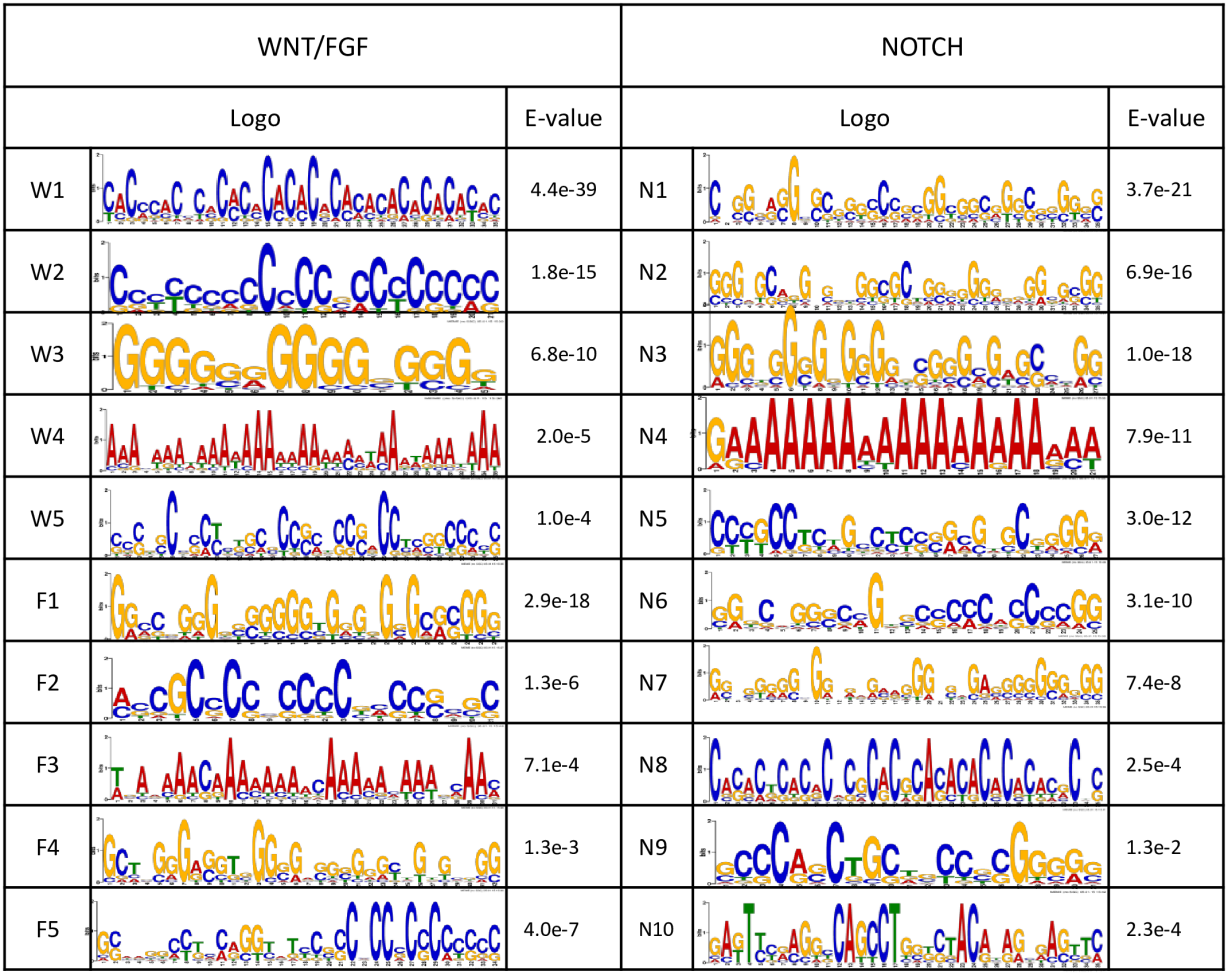
*De novo* motif discovery led to 20 statistically significant motifs overrepresented in the promoter regions of mouse cyclic genes. We found 10 motifs (named *N1-N10*) in the promoter region of Notch cyclic genes, 5 motifs for Wnt cyclic genes (*W1-W5*) and Fgf cyclic genes (*F1-F5*). The motifs were ruled significant after passing all steps described in the Methods section.

We found 5 motifs (*W*_*i*_, *i = 1.5*) significantly enriched in the promoter regions of Wnt-cyclic genes. The motifs are between 15 and 35 bp wide and every motif was present in at least 71% of the genes (10 out of 14). For Notch there are 10 significantly enriched motifs (*N1-N10*), 21 to 35 bps long, with at least 70% representation and E-value < 0.05. Finally, 5 motifs (*F1-F5*), 21 to 34 bps long, were found to be significant in the promoter regions of Fgf-cyclic genes (Fig 5) with E-value <0.05 and 70% representation. Although we used the entire probe sets present in the mouse microarray chip as the background model during the Expectation Maximization step in MEME, we took supplementary precautions to ensure that none of these motifs is a Transposable Elements (TE), by comparing them individually to the database of known TE in the eukaryote genome using Dfam [42]. Indeed, a large majority of the mouse genome is made of TEs and a blind search of motifs may be misleading.

Using the set of all published mouse cyclic genes, we generated a reference list of motifs by finding conserved regulatory elements upstream of their sequences (S1 Fig). The list, containing 10 shared motifs annotated as A1 to A10, was used to discriminate the pathways and uncover motifs specific to each of them.

We used a two-way comparison method to classify the motifs. First, we compared motifs of each pathway to the reference list using the TOMTOM tool of MEME [43]. As the reference list contains few motifs and may lead to inaccurate p-values, we took additional precautions by setting a correlation coefficient threshold and a minimum sequence overlap to reinforce the motif similarity search. Two motifs were deemed similar in the first step if the TOMTOM E-value was less than 10^−5^, the Pearson correlation coefficient > 0.6, and they had at least 60% overlap. Second, we used the MAST tool [44] of MEME to search for overrepresentation of the corresponding motif in the promoter of all known mouse cyclic genes. They were then classified as one-, two- or three-pathway motifs according to the enrichment. After passing the first step, a motif is considered as one-pathway if it is present in at least 60% of the known gene of one pathway and less than 20% of the others. A two-pathway motif must be present in at least 60% of the known genes of each of the two pathways and less than 20% of the third. Finally, a three-pathway motif is present in at least 60% of all known genes in all pathways.

**Fig 6:**
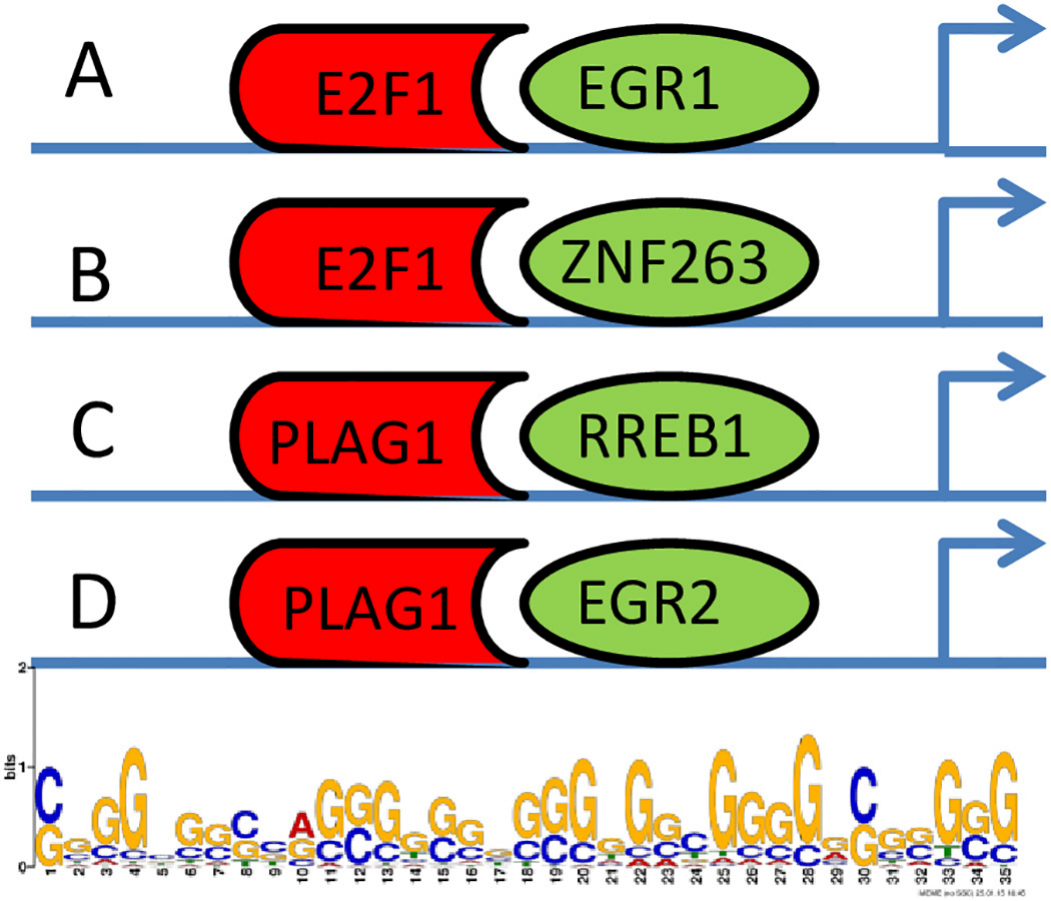
Several TFs may cooperatively regulate the expression of cyclic genes during mouse somitogenesis. A GC rich regulatory motif denoted as A1 in the text was found to be overrepresented in the promoter of nearly ¾ of reported cyclic genes during chicken somitogenesis. The average position of the motif is around 300 bps upstream of the transcription start site for mouse genes. When tested for enrichment in the database of experimentally validated TFBSs, it appears this regulatory motif may contain the binding sites of several couple of TFs acting cooperatively to regulate the expression of corresponding genes. The first and second possibilities (panels A and B) is the TF E2F1 binding upstream of EGR1 and ZNF263 respectively and the others possibilities (panels C and D) involve the TF PLAG1 binding upstream of RREB1 and EGR2.

As an example following the previous criteria, we found that the motifs *F1*, *W2*, and *N1* were all similar and corresponded to the reference motif *A1* (see S1 Fig) which is then a three-pathway motif. The motif *A1*, a 35 bp sequence with the consensus *CGGGCGGCCAGGGGGGGGGGGGGCGGGGGCGGGGG*, is also present in 594 mouse gene promoters including 95% (37 out of 39) experimentally reported mouse cyclic genes (see Table 3). A GO enrichment analysis using all 594 target genes revealed they are mostly connected to the developmental process (S2 Fig). A search in the database of known TFBSs generated many hypotheses and led to the suggestion that this motif may be the binding site of two co-transcription factors. Indeed, we found several pairs of known TFBSs in the *JASPAR CORE 2014* for vertebrates [45] significantly similar to the motif (E-value < 10–5). *The JASPAR CORE* database, which contains a curated, non-redundant set of profiles, derived from published collections of experimentally defined TFBSs for eukaryotes, is widely used as experimental validation of *de novo* motif findings. Given that the motif represents the *cis*-regulatory element of the associated genes, their regulation may be controlled by the transcription factors E2F1/PLAG1 binding upstream of EGR1/RREB1, E2F1 binding upstream of ZNF263, or PLAG1 binding upstream of EGR2 as described in Fig 6. Table 4 summarizes the discovered motifs, the possible corresponding TFs, the best possible sequence match, the GO enrichment for the target genes in the mouse genome, their connection with the Wnt, Notch or Fgf pathways, and their presence in chicken and zebrafish. The N6 and N9 Notch-related motifs were not found to be similar to any of the reference motifs. It appears that 50% of the regulatory motifs have known binding sites in the database of TFBSs and represent leading candidates for experimental investigation. Moreover, they are three-pathway motifs whose target genes are more involved in development. The regulatory motifs with no known TFBSs, are also important for uncovering the regulatory process of vertebrate somitogenesis.

**Table 4:** *De novo* identified motifs and their conservation between pathways and species. We tested the reference motif list for pathway conservation and between species conservation. For each motif, we computed the enrichment in known TFBSs databases and reported the corresponding significant TFs. It appears that several TFs may be acting cooperatively to regulate the expression of cyclic genes. We also noticed motif conservation between mouse and chicken as opposed to zebrafish where any of the discovered motifs could be found in the promoters of known cyclic genes. The conservation between species is given in terms of percentage of reported cyclic genes of the species with the corresponding motif in their promoter region.

Motifs	Possible TFs	Best possible match (5’-3’)	Related GO terms (Biological Process)	Wnt- Notch- Fgf	chicken	Zebrafish
**A1**	(E2F1/PLAG1)—(EGR1/ RREB1); E2F1—ZNF263; PLAG1—EGR2	CGGGCGGCCAGGGGGGGGGGGGGCGGGGGCGGGGG	Multicellular organismal development; Developmental process; System development	W2-N1-F1	Yes (74%)	No
**A2**	-	CACCCACGCACACACACGCACACACACACACACCC	Cellular developmental process; Anatomical structure development; Multicellular organismal development	W1-N8-**	No	No
**A3**	SP2, PREB1, ZNF263, EGR1, E2F3, EGR2	CCCCCCCCCCCCCCCCCCCGC	Anatomical structure morphogenesis; Chordate embryonic development; Organ morphogenesis	W3-N2-F4	Yes (63%)	No
**A4**	-	AAAAAAAAAAAAAAAAAAAAAAACCAACA	Primary metabolic process; Metabolic process; Cellular metabolic process	W4-N4-F3	No	No
**A5**	SP2, EGR1, PREB1, SP1, EGR2	CCCCCACCCCCCCCCCGCCCC	Developmental process; Multicellular organismal development; Anatomical structure development	W2-N3-F2	Yes (58%)	No
**A6**	-	CCGCCCCCGGGAGGCGGAGGCAGGCG	Cellular protein metabolic process; Protein metabolic process; Cellular macromolecule metabolic process	**-N5-**	No	No
**A7**	-	CCCTCGCTCTGCAGGCCGGGCTGGCCCCC	RNA processing; Metabolic process; Cellular metabolic process	W5-N10-F1	No	No
**A8**	(ZNFF263/E2F6)—EGR1	GCGGGCGGGAGGGGGGGAGAGGGGGAGAG	Neuron projection development; Cell development; Neuron projection morphogenesis	W2-N7-F5	Yes (50%)	No
**A9**	-	CGCGCGGTCTCCCCGGGGCGCCCTGGCCG	Cellular metabolic process; RNA metabolic process; Primary metabolic process	W5-**-**	Yes (50%)	No
**A10**	SP2, PREB1, EGR1	CCCCCCCCACCCCCC	Forebrain development; Central nervous system development; Brain development	W2-N3-F2	No	No
**N6**	-	GGGCAGGGCCGTGCCCCCACCCCGG	Anatomical structure development; Negative regulation of RNA metabolic process; Cellular macromolecule metabolic process	**-N6-**	No	No
**N9**	Tcf3, NHLH1, Ets1	GCCCAGCTGCTGCCCCGGGGG	System development; Negative regulation of transcription from RNA polymerase II promoter; Negative regulation of transcription, DNA-dependent	**-N9-**	No	No

To check whether the GC-rich motif A1 is specific for the somitogenesis genes or also related to the cell cycle, we have computed the enrichment of the GC-motif in the set of known cell cycle genes. We have used the promoter sequences of the cell cycle genes reported by [46] and used MAST to compute a possible enrichment of the motif A1. From the 1134 genes identified by the authors to periodically expressed during cell cycle, 112 (list in S5 Table) were found to have this motif in their promoters. This low overlap (~10%) demonstrates that the GC-rich motif is not significant in the cell cycle; the finding is in agreement with previous results in that the cell cycle and the periodic activation of gene expression during somitogenesis are two separate and independent processes.

The significant enrichment of long, guanine-rich motifs in genes regulated during somitogenesis, raises the possibility of a G-quadruplex (G4) DNA structure playing a role in the promoter region of these genes. G4 is a four-stranded nucleic acid structure formed by guanine-rich sequences. While the function of the G-quadruplex is still a topic under discussion, evidence points to a role of G4 DNA in different cellular contexts [47] [48, 49]. We investigated the prevalence of quadruplex-forming DNA sequences in the mouse, chick, and zebrafish genes periodically regulated during somitogenesis (see Methods), and in their human orthologs (Table 5). The genes transcriptionally regulated during somitogenesis are significantly enriched in G4 sequences in the mouse. Indeed 85% of known mouse cyclic genes contain sequences potentially forming G4 structures (Fisher Exact test p-value <0.007), suggesting a possible role during mouse somitogenesis. A similar enrichment was observed in human (p-value < 0.035) whereas enrichment of G4 forming sequences in chicken and zebrafish was not significant. Interestingly, the regulatory function of G4 appears to be a relatively recent evolutionary invention; the quadruplex-forming sequences are highly prevalent only in mammals and certain other land vertebrates, but absent in zebrafish and invertebrates (see e.g. [50] and http://tubic.tju.edu.cn/greglist/statistics.htm). In light of our results it is therefore likely that the postulated G-quadruplex-mediated regulatory mechanism has been adopted by the WNT/NOTCH/FGF cycle only in mammals.

**Table 5:** Distribution of G-quadruplex structures in the promoter regions of mouse, chicken, zebrafish, and human genes. We computed the distribution of G4 in the promoter (2 kbps upstream of the TSS) of mouse, chicken, zebrafish, and human genes and compared to that of corresponding cyclic genes. As cyclic genes in human are not well defined, we used homologs of mouse cyclic genes for comparison purposes. G4 is significantly represented in the promoter of mouse cyclic genes. Indeed 85% of known mouse cyclic genes contain the G4 structures, suggesting a possible role during mouse somitogenesis. The p-value is computed using Fisher exact test.

Species	Mouse		Chicken		Zebrafish		Human	
	Genome	Cyclic	Genome	Cyclic	Genome	Cyclic	Genome	Homologs of mouse cyclic genes
**Number of genes**	15293	39	13487	19	10347	9	25064	39
**Genes with G4**	6761	33	6317	11	493	1	10999	29
**% of genes with G4**	44%	85%	47%	58%	5%	11%	44%	74%
**Significantly different**	Yes		No		No		Yes	
**P-value**	0.006966		-		-		0.034693	

### 2.7. Families of TFs potentially involved in the regulation of mouse cyclic genes

Using *de novo* motif finding tools, we found a set of motifs that may help explain the gene’s regulation during mouse somitogenesis. These regulatory motifs, which are binding sites for TFs, are separated into three categories depending on whether they are conserved between one, two, or three of the Wnt, Notch, and Fgf pathways. The length of the regulatory elements suggests that some may be binding sites of several TFs acting cooperatively to recruit the RNA polymerase II and initiate transcription of the corresponding genes at a specific time during the somite cycle.

We found several families of TFs whose binding sites are overrepresented in the promoter region of reported mouse cyclic genes. Of these families, E2F, EGR, and SP are predominant.

E2F is a family of eight transcription factors that have been shown to play a crucial role in the control of cell cycle and proliferation. Their representation (E2F1, E2F2, and E2F3) in this case suggests they may be acting as activator, as these TFs, unlike other members of the family, are known to be transcriptional activators.

EGR (Early Growth Response) is a family of zinc TF factors that play a role in the development, growth control and survival of several cell types. We have observed the presence of the binding sites of two members of the EGR family (EGR1, EGR2) in the promoter region of reported mouse cyclic genes.

The GC rich regulatory motif A1 described earlier was found to be overrepresented in the promoter of nearly ¾ of known chicken cyclic genes (see Table 4). When tested for enrichment in the database of experimentally validated TFBSs, we found that this motif may be the binding of TFs acting cooperatively to regulate the expression of corresponding genes, giving rise to several possible combinations as described in Fig 6. EGR1 TF binding downstream of E2F1, in which Egr1 abrogates the block in differentiation caused by deregulation of E2F1, has already been reported [51], but it has never been reported to play any role in setting the oscillation of gene expression in vertebrates.

Table 4 summarizes the discovered regulatory motifs, their consensus sequence, the most significant GO terms associated with all genes in the mouse genome with this motif in their promoter, the conservation between pathways, and the between species conservation.

### 2.8. Regulatory elements conserved between mouse and chicken

We tested whether reported cyclic genes from mouse, chicken, and zebrafish share the same regulatory elements in their promoter. To this end, we determined if any of the discovered mouse motifs were significantly overrepresented in the promoter of chicken and zebrafish cyclic genes. An alternative to this method is to directly perform *de novo* motif finding using cyclic genes of these species. But unlike in the mouse, the number of cyclic genes in the chicken and zebrafish is very limited and insufficient for *de novo* motif finding.

As summarized in Table 4, 50% of the regulatory motifs are conserved between the mouse and chicken, and there is no conservation between the mouse and zebrafish. The motif A1 described earlier as a possible binding site for E2F1 and EGR1 acting cooperatively, was found in nearly ¾ of reported chicken cyclic genes. Other conserved motifs include A3 whose length suggests it could be the binding site of a combination of TF including SP2, PREB1, ZNF263, EGR1, E2F3, and EGR2.

### 2.9. Regulatory motifs in bimodal genes

The timing and analysis of expression profiles during mouse somitogenesis in our previous study [15] led to the list of new candidate cyclic genes with one or two peaks of expression. We checked whether the regulatory motifs discovered earlier may be present in the promoter of these genes, as this may reinforce their case as strong candidate cyclic genes. To this end, we used the MAST tool to compute enrichment of the reference motifs in their promoter, as well as the promoter of the chicken and zebrafish candidate cyclic genes proposed in this study. Overall, it appears that 151 out of 164 proposed candidate cyclic genes in the mouse contain at least one reference motif in their promoter with E-value < 10^−5^ (corresponding to a lower p-value). The number of genes containing a particular motif is listed in Table 5, as well as those most significant according to their E-values. As these genes were selected based on the amplitude of their LS periodogram (measure of the periodicity), and the regularity of their profile, the presence of the reference motifs makes them even stronger candidate cyclic genes.

Some reference motifs were also found to be significantly overrepresented in the promoter on bimodal genes. 101 candidate bimodal genes contain at least one motif, 97 of which contain a combination of two motifs or more.

Tested against the reference list of motifs for enrichment, the candidate cyclic genes with one or two peaks of expression in the chicken dataset showed some significance whereas the candidate genes for zebrafish showed no significance at all (see Table 6). This confirms our earlier observation that there is no regulatory motif conservation between mouse and zebrafish cyclic genes.

**Table 6:** Motif enrichment in the lists of candidate cyclic genes from mouse, chicken, and zebrafish datasets. We have compiled the lists of candidate cyclic genes with one and two peak of expression during mouse, chicken, and zebrafish somitogenesis and tested them against the reference of regulatory motifs. For each species, we reported the number of genes containing the corresponding motif in their promoter as well as the genes with the highest E-values. These genes represent leading cyclic candidate genes. As expected, none of the motifs discovered in mouse was found to be overrepresented in the promoter of zebrafish candidate cyclic genes.

Motifs	Mouse unimodal [15] (164 genes)	Mouse bimodal [15] (173 genes)	Chicken unimodal (S1 Table, 211 genes)	Chicken bimodal (S2 Table, 131 genes)	Zebrafish unimodal (S3 Table, 111 genes)	Zebrafish bimodal (S4 Table, 61 genes)
**A1**	(87 genes): Cmtm6, Ptpn11, Fgfrl1, Pole, Ccnd1, Skil, Llf3	(52 genes): Fgf13, Wsb2, Reep5 Pik3ca, Arfip2, Ndfip1	(90 genes): PSMB7, PCSK5, PTRH2, MAP2K6	(50 genes): WBSCR22, SAMD15	0	0
**A2**	(52 genes): Start3, Psmd7, Timm44, Lrig3, Mark1, Ephb4	(41 genes): Ddah2, Cdkn1a, Rnpep, Scand1, Bola2, Vps37a	(5 genes): FBLN2, MNAT1, CDC42BPA	(1 gene): ESD	0	0
**A3**	(60 genes): Cmtm6, Safb2, Fgfrl1, Ankhd1, Msi2, Ccnd1	(31 genes): Reep5, Rcor1, Creb3, Ube3b, Tcap, Isca2	(38 genes): MAP2K6, EPC2, URM1, OLA1	(1 gene): RNF126	0	0
**A4**	(85 genes): Ankhd1, Fam13c, Akap1, Mtm1, Nova1, Ogt, Thra	(72 genes): Oxa1l, Pik3ca, Rnpep, Csnk2a1, Antxr1, Surf4	(10 genes): SHPK, MAD2L1, OLA1, WBP4	(7 genes): RARB, FADD	0	0
**A5**	(43 genes): Safb2, Cmtm6, Ccnd1, Fgfrl1, Qser1, Akirin1	(35 genes): Fgf13, Vps37a, Zfp219, Tcap, Kap, Gnb1	(54 genes): PSMB7, MAP2K6, CEP76, XPO7	0	0	0
**A6**	(62 genes): Tnpo3, Ube2l3, Urm1, Efemp2, Slc16a11, Trub2	(45 genes): Ndfip1, Grn, Reep5, Rad17, Tor1b, Cdkn1a	0	0	0	0
**A7**	(61 genes): Rps9, Rbm14, Paf1, Tnpo3, Ddx46, Slc16a11, Zfp7	(46 genes): Phf20, Shmt1, Rab22a, Rad17, Tor1b, Vcp	0	0	0	0
**A8**	(55 genes): Coro2b, Hsd17b7, Dnajc3, Cmtm6, Ugp2, Ephb4	(41 genes): Spred2, 9030624J02Rik, Tcap, Copg2, Dgat2	(35 genes): FAR1, MRPL32, PTRH2, CTNND1	(2 genes): MEF2BNB, CCNK	0	0
**A9**	(46 genes): Wdr33, Akap1, Timm44, Tnpo3, Ddx46, Trub2	(39 genes): Tor1b, Zc3h11a, Med1, Gnpda2, Ddah2, Suz12	(3 genes): SEC14L1, PEX16, PIGW	0	0	0
**A10**	(41 genes): Wdr33, Fgfrl1, Ercc8, Safb2, Hdlbp, Thra, Exosc4, Coro2b	(28 genes): Add3, Tcap, Reep5, Creb3, Kap	0	0	0	0

### Conclusions

We used the previously developed Maximum Entropy deconvolution method to estimate the time at which peaks of gene expression are observed during zebrafish and chicken somitogenesis, and compared the timelines with the mouse. We have confirmed the existence of genes with two peaks of expression during one somite cycle. Although the specific function of such bimodal regulation has not been established yet for this class of genes, one possibility is that they may be involved in synchronizing the regulation between the opposite phases of the cycle. Although subsequent experimental data are not available yet to infer all putative causations in the chick and zebrafish somitogenesis, the timelines of gene expression presented in this study constitute a valuable resource that may be used for generating informed hypotheses concerning causal relations between the genes involved in somite formation. These relations may be used in studies of somitogenesis, but also of other processes that involve time-dependent transcriptional regulation of genes from these pathways, one prominent example is the Epithelial-to-Mesenchymal Transition, e.g.[52].

At the molecular level, we identified several regulatory motifs, upstream of mouse cyclic genes, which may be binding sites for TFs regulating oscillatory gene expression during somite development. The regulatory motifs were then classified as one-, two-, or three-pathway motifs depending on whether they are specific to one, two or three pathways respectively. The bimodal genes contain mostly two-pathway motifs, which supports the hypothesis that they may serve as a linker between pathways. We also found that promoters of somitogenesis cyclic genes in the mouse are significantly enriched in G-quadruplex structures as compared to the whole mouse genome. Indeed, 33 out of 39 known mouse cyclic genes contain at least one G-quadruplex forming sequence.

## Methods

### 3.1. Data preprocessing

As the primary expression dataset, we used the gene expression data of [8]. These data, available from ArrayExpress under accession E-MTAB-406, are genome-wide relative mRNA expression levels in the tails of 18 and 21 embryos at different stages of the oscillation generating a new somite during chicken and zebrafish somitogenesis, respectively. The *CEL* files were downloaded and preprocessed using the *Robust Multi-Array* (RMA) function of the “affy” package of Bioconductor [53]. As a result, we created a normalized and background corrected set of expression values that was subsequently summarized for each species. Additional data filtering included the present-absent detection check (only probe sets called “present” in at least one half of the total arrays were retained), the peak-to-through ratio (minimum cutoff set at 1.7) which led to 13717 probe sets (35.6% of the probe sets present on the chip) and 8729 probe sets (55.9% of the probe sets present on the chip) for the chicken and zebrafish dataset respectively. The subsequent datasets represent mRNA levels for each time point (or embryo) running over one somite cycle. These filtering steps have been shown to reduce the number of false positives on microarray data and were recently used to precisely predict gene expression profiles after burn injury [54].

For each dataset, we successively applied the LS algorithm to rank the probe sets according to their periodicity, the maximum entropy deconvolution to extract the individual profile of each probe set, and determine the accurate timing as well as corresponding errors. At 95% confidence, 263 probe sets from de chick dataset were found to be statistically significant, as measured by their Lomb-Scargle periodogram (LS p-value < 0.05 and fdr < 0.1), and thus were ruled as potential cyclic genes. We then applied the Maximum Entropy deconvolution algorithm to the resulting probe sets to extract the individual profile of each gene, and estimate the timing as well as its resolution. Final lists of oscillating genes were established based on the regularity of the profile and the high resolution of the timing. After manual inspection of the profiles, 211 probe sets, each with a regular profile and timing error less than 10 minutes, were selected. To identify genes with two peak of expression in chick, we applied the LS test at double frequency. 141 probe sets were found to be statistically significant (p-value<0.05, the probability that the bimodality is indeed due by chance and the false discovery rate -fdr- <0.1). For these probe sets, the Maximum Entropy deconvolution and manual inspection of expression profiles led to 84 probe sets with two peaks of expression.

The same method and statistics were applied to the zebrafish dataset leading to 8729 high quality probe sets, 116 of which showed an oscillatory pattern with one peak (LS p-value < 0.05 and fdr < 0.15). We then extracted the individual profiles, computed the timing, and estimated the accuracy of these probe sets, after which, 111 with estimated timing error of no more than 5 minutes were selected and ruled as candidate cyclic genes with one peak of expression. In addition, we identified 65 probe sets with two peaks of expression per cycle (LS p-value < 0.05 and fdr < 0.1). After filtering for peak regularity and timing accuracy, we identified 32 of these as candidate cyclic genes with two peaks of expression during zebrafish somitogenesis.

### 3.2. Accurate cycle phase for collected data points

One major challenge when studying somitogenesis using genome profiling methods is data collection. As all information must be derived from the embryo itself, the study of genetic change over one somite cycle implies the sacrifice of several embryos, making the process impossible to study in humans. In general, these embryos are aligned retrospectively according to the fluorescence of the reference cyclic genes along the PSM. Instead of assuming evenly spaced times between embryos along the clock cycle, we have developed a method, based on the optimization of the Lomb-Scargle (LS) amplitude, which assign times to measurements such that the expression of the set of known cyclic genes are the most periodic, as measured by the amplitude of the best-fit harmonic wave [15]. *her1*, *her7*, *dlc*, and *tbx16* have been shown to be cyclic during zebrafish somitogenesis and represent good candidates for phase optimization. For the chicken phase optimization, we used *T*, *LFNG*, *HES5*, *HES1*, *NKD1*, *HEY1*, and *DACT1* which have been shown to have oscillatory expression patterns during somites formation (see Table 3 for references).

**Fig 7:**
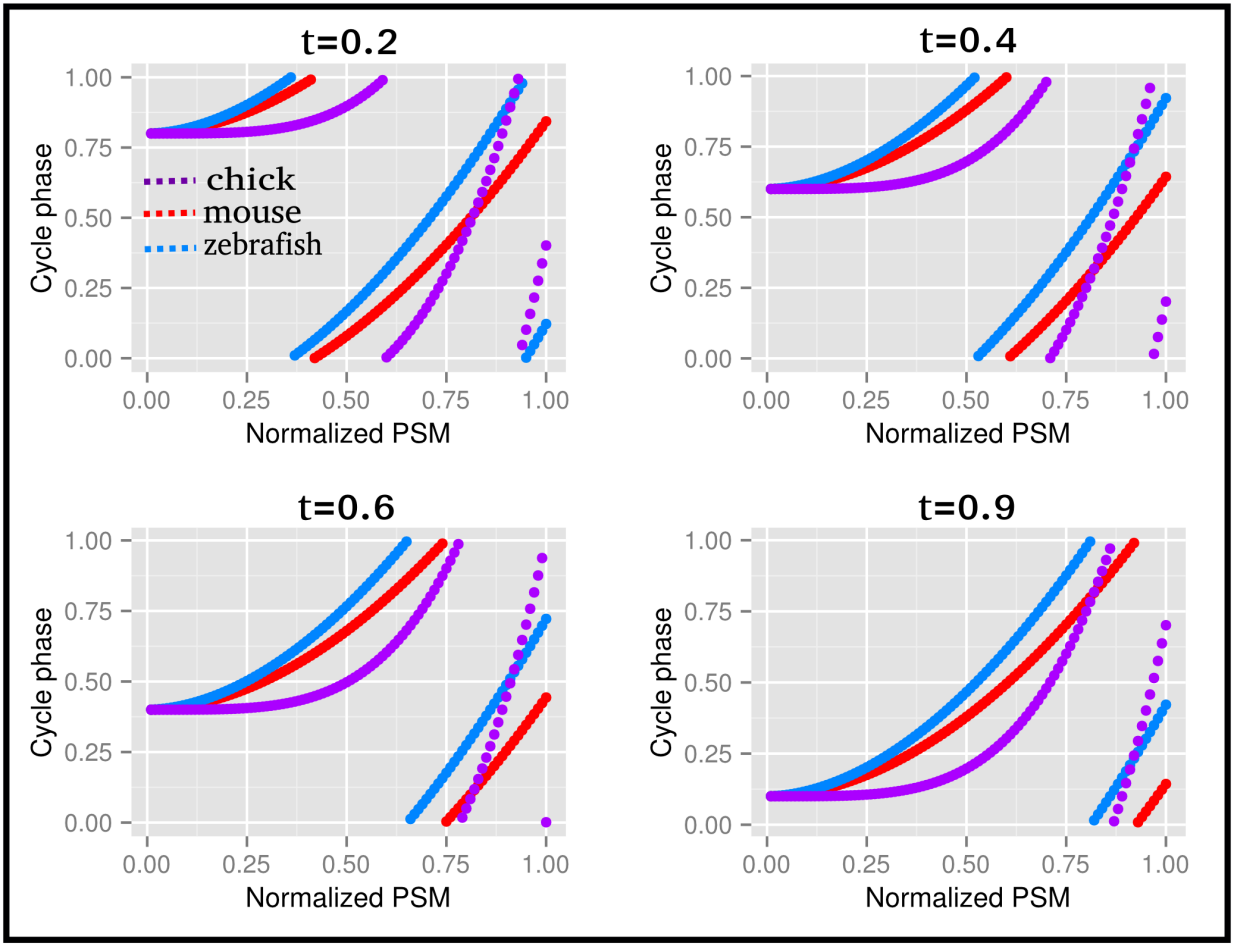
Relationship between the phase of gene expression and position along the PSM of the chicken, mouse and zebrafish embryos. The waves start as moving fast for small values of *x* (most posterior part of the PSM), and slow down as the wave progresses toward larger values of *x*. The four snapshots depict a sequence of time-points, corresponding to different phases of the somite cycle.

As described in [15], the studied sample always contains cells in different stages of the cycle, thus affecting the observed temporal changes in gene expression. We used a formula derived from the wave propagation in physics to describe the dependence between the position of the reference cyclic gene band *x*, the time *t* and the phase *φ*. At a given time and phase, the position of the reference gene is given by *x*(*φ*, *t*) = (1 − d)(*φ* + *t*)^1/*α*^. The parameters *α* and *d*, which describe the wave deceleration and geometry of the system are computed as in [15]. As explained in [4, 8], the reference cyclic genes are *Lfng* for the mouse and chicken, and *Her7* for the zebrafish. The position of the highest density of the reference gene transcript for each species was measured by analyzing the In-Situ Hybridization (ISH) images provided by the authors. The computed values of *d* and *α* are presented in Table 7, and Fig 7 depicts the relationship between wave propagation and the position of the reference gene along the PSM.

**Table 7:** Parameters describing the wave deceleration and the geometry of the system for collected data in mouse, chicken and zebrafish somitogenesis. The parameters *α* and *d* are computed assuming that the cycle phase *φ* is random at the time when the embryos are sacrificed [15].

	Mouse [15]	Chick	Zebrafish
***d***	0.022	0.110	0.140
***α***	1.900	4.040	1.850

The homology between species was defined using the current (build68 updated on 04/15/2014) version of the homologene dataset containing curated list of genes and orthologous from different species from NCBI (http://www.ncbi.nlm.nih.gov/homologene). For each gene, the corresponding homolog was found using the species taxonomy as well as the gene id.

### 3.3. Timing of gene expression

The kernel functions obtained in the previous section were used for the spatiotemporal maximum entropy deconvolution of gene expression profiles for each species. For every profile, we applied the peak detection algorithm and estimated the accuracy of the timing using a Monte Carlo scheme. The algorithms used in this section are extensively described in [15].

### 3.4. Validation of regulated genes

Analyzing the whole-genome expression data provides evidence for oscillatory expression pattern in a number of genes that were previously not annotated as transcriptionally regulated during somitogenesis because the p-value threshold was not satisfied in the scoring methods used in these studies [4, 8]. Detecting oscillatory gene expression using our method provides an important benchmark for future experimental analyses. Indeed, the list of genes with very low LS p-value (p<0.01) and highly regular profiles detected in mouse contains 95% of the known cyclic genes. For selected genes, independent evidence pointing to spatiotemporal regulation during somitogenesis has been collected by analyzing the plates available in the *e-mouseatlas* resource (http://www.emouseatlas.org/emage/) [55]. E-mouseatlas contains FISH (fluorescent in-situ hybridization) images of mice at different stages of their development, including somitogenesis. FISH images are available for known cyclic genes like *Axin2* (EMAGE:4670), *Nrarp* (EMAGE:4221), *Dkk1* (EMAGE:3868), *Hes1* (EMAGE:3694) as well as candidate cyclic genes with one peak of expression, e.g. *Kef10* (EMAGE:2256), *Fscn1* (EMAGE:4607) and candidate cyclic genes with two peak of expression including *Med20* (EMAGE:26452), *Ciao1* (EMAGE:1554) which present sequential regulation in the tail-bud. While precise timing cannot be derived from these images, the differential activation of these genes in the tail-bud and PSM is observed in the e-mouseatlas images, which is consistent with a possible oscillation pattern and thus constitutes additional evidence supporting the involvement of these genes in the somite cycle.

### 3.5. Gene Ontology enrichment analysis

Gene Ontology analysis was performed using the DAVID (Database for Annotation, Visualization and Integrated Discovery, http://david.abcc.ncifcrf.gov/) web server. For each species and sub-list, we uploaded the gene list to the server and set the background as the total number of genes present in the corresponding microarray chip. Moreover, only GO terms containing at least 50% of the input genes and a q-value (Benjamini corrected p-value) < 0.05 were selected. We subsequently used The REViGO (Reduce + Visualize Gene Ontology, http://revigo.irb.hr/) [56] server to reduce the redundancy in the GO lists.

### 3.6. *De novo* motif identification

Regulatory motifs are short, usually fixed-length nucleotide sequences commonly found upstream of genes whose expression they control. They can represent TFBSs, splice junctions and binding domains. Discovering these motifs and the related TFs that bind them may lead to a better understanding of the transcriptional regulation of gene expression [57, 58]. In the case of somite formation where several pathways are believed to be involved, determining the set of TFs either specific to each pathway and each time interval or common to all genes involved in the process will help building the regulatory network and cross-talk between the Wnt, Notch and Fgf pathways.

Identification of regulatory motifs was performed following several steps. First, for each pathway we downloaded the promoter sequences of the corresponding genes using the package “*biomaRt*” [59] from the Bioconductor platform (with the genome version GRCm38.p1 for mouse, Zv9 for zebrafish and Galgal4 for chicken). In this study, we successively considered 1kb, 2kb, 5kb and 10kb upstream of the transcription start site of the gene as the promoter of the gene and subsequent analysis led to the conclusion that 2kb is more suitable as the promoter length in this particular case. It has been shown [43, 60–62] that many motif discovery algorithms perform badly in the presence of low-complexity DNA, tandem repeats, SINES, and ALUs with the resulting motifs mainly composed of repetitive elements (RE). To eliminate the RE to the list of motifs, all discovered motifs were uploaded to the DFAM database to be compared against the list of known Repetitive elements in eukaryote genomes [42] and those containing RE were removed from the list. We then used the MEME tools [27] (online and local stand-alone version) for *de novo* motif finding, motif comparison and enrichment in other databases.

We ran MEME using a 2-order Markov background correction computed using the promoter sequences of the mouse genome excluding all mouse cyclic genes. Only motifs with E-value < 0.05 were considered significant. We subsequently used MAST to search other sequence databases for the occurrence of discovered motifs and TOMTOM to find similar motifs in experimentally validated databases such as JASPAR. The presence of a motif as DNA-binding preference in JASPAR gives a strong indication of reliability as this database is composed of experimentally and manually curated TFBSs. To identify possible roles for the discovered motifs, we used GOMO (Gene Ontology Motif Enrichment) and reported only GO terms with a p-value < 10^−5^ and at least 50% specificity.

It should be noted that inferring regulatory elements functionally significant in a set of genes is complex as there is no a priori optimal method to infer the position of the motif with respect to the gene. In general, the promoter length in eukaryotes can be anywhere between several hundred and ten thousand base pairs or more. In this study, we undertook a global approach by testing the motif enrichment using 1kb, 2kb, 5kb and 10 kb of upstream genomic sequence. We subsequently chose the 2kb sequences as most representative of the promoter region. The rationale is that the 2kb analysis turned out to be the best compromise between false positives (high for longer sequences) and false negative (high for too short sequences) ratios. Specifically, the 2kb promoter length gives better accuracy and the discovered motifs where found in more than 90% of the known cyclic gene set (see S6 Table). Indeed, the 1 kb analysis provided significant hits with E-value < 10–30, but with low gene coverage compare to 2kb (on average, only 61.2% of known cyclic genes contained at least one of the discovered motifs). This may not be useful as the reference motifs must be present in most genes.

The 5kb and 10 kb analysis provided longer motifs that are more often associations of motifs found with the 2kb analysis and lower gene coverage. Nonetheless, using the 5kbp or 10kbp promoter sequences did not yield any significant overlap between mouse and zebrafish.

### 3.7. Predicting G-Quadruplexes

To identify potential G-quadruplex DNA structures (derived from one strand of genomic DNA), we used the Quadparser application [63] with the standard options (sequence of three or more G or C bases repeated four times, each separated by loops 1.7 base pairs long). We predicted the G4 DNA-forming sequences in 2 kbps upstream of coding sequence for every gene in the mouse, chicken, zebrafish, and human based on the GRCm38.p1, Galgal4, Zv9, and GRCh37.p11 genome annotations, respectively.

## Supporting Information

S1 Fig Regulatory motifs overrepresented in the promoters of mouse cyclic genes. *De novo* motifs finding led to 10 statistically significant motifs overrepresented in the promoter regions of all mouse cyclic genes. These motifs were categorized as one-, two-, or three-pathway motifs according to their overrepresentation in Wnt, Notch, and Fgf pathways (see text).
(TIF)

S2 Fig Genes containing the motif A1 in their promoter are involved in developmental process. Ttreemap visualization obtained by REViGO analysis of the summary of GO Biological Process of the 594 promoters of mouse genes, including 95% of known cyclic, containing the GC-rich motif, A1. The dimension of the area is proportional the p-value and the color similarity denotes semantic similarity between terms.
(TIFF)

S1 Table The list of genes with one peak of expression during chicken somitogenesis. The timing of genes found with one peak of expression during chicken somitogenesis, ranked according to their LS p-value and the regularity of the profile. Times in minutes assume a 90mn periodicity for every transcript and errors are computed by adding to the original transcript source of noise typically found in microarray experiments.
(DOCX)

S2 Table The list of genes with two peaks of expression during chicken somitogenesis. The timing of genes found with two peaks of expression during chicken somitogenesis, ranked according to their LS p-value and the regularity of the profile. Times in minutes assume a 90mn periodicity for every transcript and errors are computed by adding to the original transcript source of noise typically found in microarray experiments.
(DOCX)

S3 Table The list of genes with one peak of expression during zebrafish somitogenesis. The timing of genes found with one peak of expression during zebrafish somitogenesis, ranked according to their LS p-value and the regularity of the profile. Times in minutes assume a 30mn periodicity for every transcript and errors are computed by adding to the original transcript source of noise typically found in microarray experiments.
(DOCX)

S4 Table The list of genes with two peaks of expression during zebrafish somitogenesis. The timing of genes found with two peaks of expression during zebrafish somitogenesis, ranked according to their LS p-value and the regularity of the profile. Times in minutes assume a 30mn periodicity for every transcript and errors are computed by adding to the original transcript source of noise typically found in microarray experiments.
(DOCX)

S5 Table The list of known cell cycle genes enriched with the GC-rich motif A1 in their promoters. From the set of 1134 genes identified to periodically expressed during cell cycle, only 112 were found to have the GC-rich motif A1 in their promoters. This low overlap (~10%) demonstrates that the GC-rich motif is not significant in the cell cycle, in agreement with previous results in that the cell cycle and the periodic activation of gene expression during somitogenesis are two separate and independent processes.
(DOCX)

S6 Table Influence of the length of the promoter on the motif analysis of cyclic genes. We compare the statistics of the motif analysis for promoter lengths of 1kb, 2kb, 5kb and 10 kb. The 2kb analysis appears to be the best compromise between false positives (high for longer sequences) and false negative (high for too short sequences) and provide better gene coverage.
(DOCX)

## Abbreviations

PSM: Presomitic Mesoderm
TSS: Transcription Start Site
TFBS: Transcription Factor Binding Site
TF: Transcription Factor
GEF: Guanine nucleotide Exchange Factors
LS: Lomb-Scargle
GO: Gene Ontology
TE: Transposable Element
