# Supplementary figures and images for: Comparison between Timelines of Transcriptional Regulation in Mammals, Birds, and Teleost Fish Somitogenesis
Source: PLoS One. 2016 May 18;11(5):e0155802. doi: 10.1371/journal.pone.0155802 (PMC4871587; doi:10.1371/journal.pone.0155802)

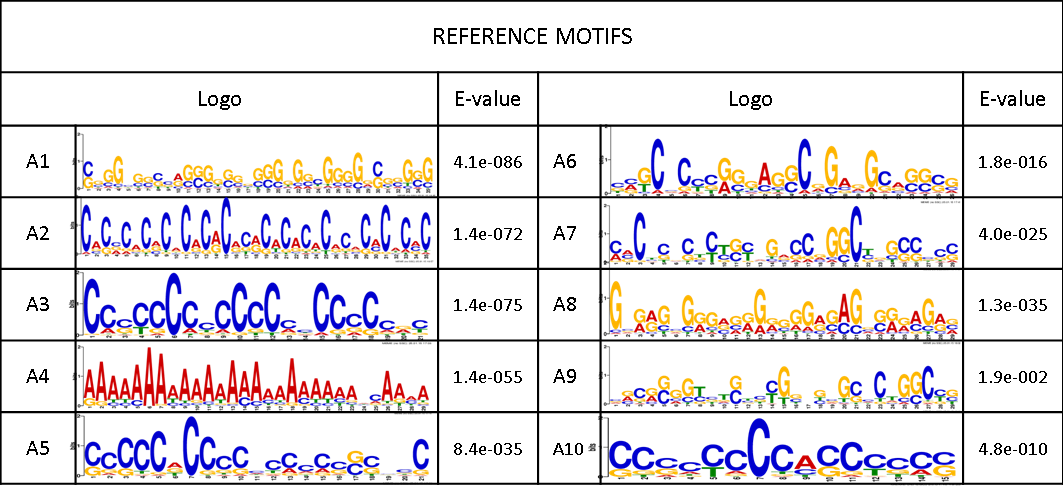

Supplement: S1 Fig — De novo motifs finding led to 10 statistically significant motifs overrepresented in the promoter regions of all mouse cyclic genes. These motifs were categorized as one-, two-, or three-pathway motifs according to their overrepresentation in Wnt, Notch, and Fgf pathways (see text). (TIF) [file pone.0155802.s001.tif]

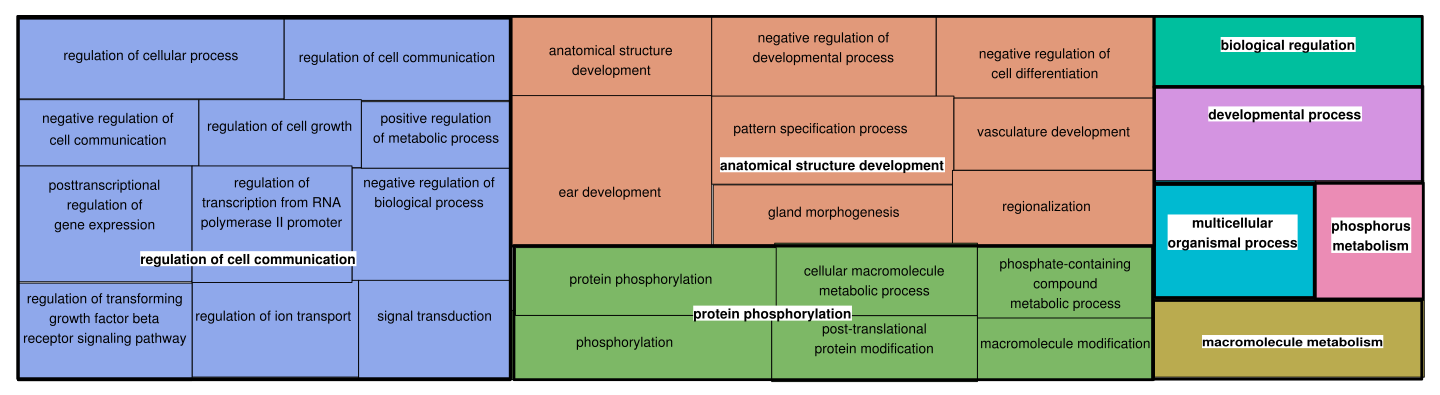

Supplement: S2 Fig — Ttreemap visualization obtained by REViGO analysis of the summary of GO Biological Process of the 594 promoters of mouse genes, including 95% of known cyclic, containing the GC-rich motif, A1. The dimension of the area is proportional the p-value and the color similarity denotes semantic similarity between terms. (TIFF) [file pone.0155802.s002.tiff]
