# Supplementary material for: Comparison between Timelines of Transcriptional Regulation in Mammals, Birds, and Teleost Fish Somitogenesis
Source: PLoS One. 2016 May 18;11(5):e0155802. doi: 10.1371/journal.pone.0155802 (PMC4871587; doi:10.1371/journal.pone.0155802)
Supplement: S1 Table — The timing of genes found with one peak of expression during chicken somitogenesis, ranked according to their LS p-value and the regularity of the profile. Times in minutes assume a 90mn periodicity for every transcript and errors are computed by adding to the original transcript source of noise typically found in microarray experiments. (DOCX) [file pone.0155802.s003.docx]

**S1 Table: The list of genes with one peak of expression during chicken somitogenesis**.

| **Probe set ID** | **Gene** | **Time(min)** | **Err(min)** | **LS p-value** |
| --- | --- | --- | --- | --- |
| Gga.3180.1.S2_a_at | *LFNG* | 78 | 5 | 0.00345 |
| Gga.3754.2.S1_at | *HES1* | 72 | 4 | 0.00442 |
| Gga.11242.1.S1_at | *HES5* | 78 | 6 | 0.00477 |
| GgaAffx.23401.4.S1_s_at | *DOCK7* | 87 | 6 | 0.00531 |
| Gga.14954.1.S1_at | *NAALADL2* | 75 | 8 | 0.00615 |
| GgaAffx.7254.1.S1_at | *AZI2* | 77 | 4 | 0.00704 |
| GgaAffx.22378.1.S1_s_at | *PLCG2* | 75 | 6 | 0.00712 |
| Gga.3772.1.S1_a_at | *T* | 49 | 2 | 0.00737 |
| Gga.4514.2.S1_s_at | *RRM1* | 76 | 3 | 0.0076 |
| Gga.5407.3.S1_a_at | *TCEA2* | 82 | 2 | 0.00879 |
| Gga.19711.1.S1_at | *GOLT1B* | 77 | 4 | 0.00884 |
| GgaAffx.7045.1.S1_at | *COG2* | 78 | 5 | 0.00974 |
| GgaAffx.10108.1.S1_at | *TTK* | 80 | 2 | 0.00993 |
| GgaAffx.5847.1.S1_at | *NPY2R* | 80 | 2 | 0.00994 |
| GgaAffx.6850.1.S1_at | *BTBD7* | 74 | 8 | 0.01066 |
| GgaAffx.13087.1.S1_s_at | *NDUFA9* | 7 | 2 | 0.01143 |
| Gga.14890.1.S1_at | *GPR1* | 31 | 3 | 0.01144 |
| GgaAffx.5084.1.S1_at | *CCDC88A* | 81 | 1 | 0.01144 |
| Gga.12258.1.S1_s_at | *MRPS34* | 76 | 4 | 0.01144 |
| GgaAffx.10846.1.S1_at | *PCDH20* | 14 | 2 | 0.01169 |
| Gga.13566.1.S1_at | *UBE2L3* | 85 | 3 | 0.01172 |
| Gga.11845.1.S1_at | *ANO1* | 54 | 9 | 0.01243 |
| Gga.6131.1.S1_at | *DNAJC8* | 45 | 1 | 0.01276 |
| GgaAffx.7711.1.S1_at | *KIF18A* | 79 | 1 | 0.0128 |
| Gga.8814.1.S1_s_at | *NANS* | 75 | 5 | 0.01329 |
| GgaAffx.7693.2.S1_at | *ELP4* | 12 | 3 | 0.01341 |
| Gga.6127.1.S1_at | *ADCK3* | 46 | 1 | 0.01349 |
| GgaAffx.13028.1.S1_at | *ZBTB8B* | 44 | 6 | 0.0139 |
| GgaAffx.26190.1.S1_at | *MAP2K6* | 83 | 2 | 0.01401 |
| Gga.12240.1.S1_at | *ACSBG1* | 61 | 5 | 0.01448 |
| Gga.12626.2.S1_s_at | *HCFC2* | 75 | 2 | 0.01448 |
| Gga.8436.2.S1_a_at | *C4H4orf33* | 12 | 3 | 0.01471 |
| Gga.1785.1.S1_s_at | *WLS* | 47 | 5 | 0.01486 |
| GgaAffx.12939.1.S1_s_at | *ATF7IP* | 82 | 2 | 0.01506 |
| Gga.10123.1.S1_a_at | *IFT27* | 42 | 7 | 0.01553 |
| GgaAffx.26222.1.A1_s_at | *NCOR1* | 77 | 7 | 0.01576 |
| Gga.16431.1.S1_at | *SMCR7* | 65 | 3 | 0.01615 |
| Gga.7911.1.S1_a_at | *COPS8* | 84 | 2 | 0.0164 |
| Gga.17393.1.S1_at | *ZFC3H1* | 68 | 5 | 0.01651 |
| GgaAffx.7363.1.S1_at | *PTPN4* | 74 | 4 | 0.0168 |
| Gga.5799.1.S1_s_at | *P4HA2* | 63 | 3 | 0.01682 |
| GgaAffx.26488.1.S1_s_at | *ACTR8* | 14 | 3 | 0.01726 |
| Gga.5931.2.S1_at | *BCAS2* | 50 | 6 | 0.01729 |
| Gga.1141.1.S1_at | *MDH1* | 47 | 4 | 0.01748 |
| GgaAffx.2351.2.S1_s_at | *RORA* | 74 | 7 | 0.01775 |
| Gga.4751.1.S1_at | *RBM22* | 13 | 4 | 0.01782 |
| Gga.5470.1.S1_at | *CHMP5* | 40 | 6 | 0.01812 |
| Gga.13983.1.S1_at | *SHCBP1* | 78 | 1 | 0.01817 |
| GgaAffx.26635.2.S1_s_at | *COPG* | 76 | 5 | 0.01827 |
| GgaAffx.269.1.S1_x_at | *KLHL12* | 13 | 3 | 0.01832 |
| Gga.6232.1.S1_at | *DERL2* | 23 | 5 | 0.01834 |
| GgaAffx.10983.1.S1_at | *ZMYM2* | 76 | 5 | 0.01891 |
| GgaAffx.1027.1.S1_at | *TRAF1* | 63 | 8 | 0.01902 |
| Gga.7649.1.S1_at | *GNB1L* | 30 | 6 | 0.01928 |
| Gga.4519.2.S1_s_at | *TPD52* | 16 | 3 | 0.01941 |
| Gga.329.1.S1_at | *HAS2* | 40 | 3 | 0.0198 |
| Gga.4649.1.S1_s_at | *PSMC3* | 12 | 3 | 0.02043 |
| Gga.2543.1.S1_s_at | *REPS2* | 82 | 1 | 0.02055 |
| GgaAffx.25111.1.S1_s_at | *IPO5* | 78 | 1 | 0.02073 |
| GgaAffx.23410.1.S1_at | *ARMC8* | 79 | 2 | 0.02107 |
| Gga.12492.1.S1_at | *TMEFF1* | 77 | 2 | 0.02116 |
| Gga.6311.1.S1_at | *HEY1* | 70 | 3 | 0.02147 |
| GgaAffx.23741.1.S1_at | *DACT1* | 81 | 6 | 0.0218 |
| Gga.9974.1.S1_at | *PIGW* | 78 | 3 | 0.02196 |
| Gga.8479.1.S1_at | *FUBP1* | 64 | 5 | 0.02271 |
| Gga.13504.1.S1_at | *RRM2* | 40 | 7 | 0.0228 |
| GgaAffx.11670.1.S1_at | *EHD3* | 38 | 7 | 0.02286 |
| Gga.2000.1.S1_at | *SLIT3* | 76 | 3 | 0.023 |
| GgaAffx.3200.1.S1_s_at | *FBLN2* | 76 | 1 | 0.02303 |
| Gga.7694.1.S1_at | *MFSD7* | 74 | 5 | 0.02315 |
| GgaAffx.20992.1.S1_s_at | *PSMB7* | 71 | 6 | 0.0232 |
| Gga.633.1.S1_at | *EPHB6* | 14 | 5 | 0.02329 |
| GgaAffx.2516.1.S1_at | *TET1* | 76 | 8 | 0.02362 |
| Gga.4283.1.S1_at | *CTNNB1* | 51 | 3 | 0.02377 |
| GgaAffx.3099.1.A1_at | *URM1* | 45 | 3 | 0.02396 |
| Gga.12366.1.S1_at | *NUP37* | 75 | 5 | 0.02417 |
| Gga.1687.1.S1_at | *TMEM237* | 40 | 5 | 0.02492 |
| Gga.13220.1.S1_at | *DUSP22* | 9 | 3 | 0.02493 |
| GgaAffx.10518.1.S1_s_at | *MELK* | 72 | 3 | 0.02504 |
| GgaAffx.25847.1.S1_at | *DUS2L* | 14 | 3 | 0.02516 |
| GgaAffx.9329.3.S1_s_at | *DHX29* | 71 | 2 | 0.02517 |
| Gga.9717.2.S1_s_at | *SECISBP2* | 62 | 6 | 0.02593 |
| Gga.2701.1.S2_at | *FGF3* | 76 | 7 | 0.02595 |
| GgaAffx.7774.4.S1_s_at | *UTRN* | 79 | 5 | 0.02674 |
| Gga.2854.2.S1_a_at | *MRPL32* | 39 | 1 | 0.0269 |
| GgaAffx.3765.1.S1_s_at | *CDC7* | 84 | 3 | 0.02721 |
| Gga.6154.1.S1_at | *TMEM59L* | 50 | 5 | 0.02776 |
| GgaAffx.7903.1.S1_s_at | *EPC2* | 4 | 1 | 0.02781 |
| Gga.4449.1.S1_at | *SERBP1* | 45 | 6 | 0.02789 |
| Gga.11692.1.S1_at | *TMEM178B* | 58 | 3 | 0.02796 |
| Gga.4918.1.A1_at | *EIF4H* | 48 | 5 | 0.02796 |
| GgaAffx.313.1.S1_at | *SMEK2* | 85 | 7 | 0.02798 |
| Gga.13495.1.S1_at | *PHYH* | 27 | 2 | 0.02876 |
| Gga.6308.1.S1_s_at | *METTL2A* | 25 | 2 | 0.02877 |
| Gga.1181.1.S1_s_at | *BRP44* | 27 | 6 | 0.02917 |
| GgaAffx.23146.1.S1_s_at | *ANKHD1* | 5 | 2 | 0.02925 |
| GgaAffx.9370.1.S1_at | *PPWD1* | 68 | 6 | 0.02931 |
| GgaAffx.24921.2.S1_s_at | *TAB3* | 76 | 5 | 0.02978 |
| Gga.1302.1.S1_at | *FAM32A* | 46 | 2 | 0.0304 |
| Gga.892.1.S1_at | *ID1* | 67 | 1 | 0.03083 |
| Gga.8082.1.A1_at | *RBM45* | 22 | 7 | 0.03127 |
| Gga.8363.1.S2_at | *AXIN2* | 21 | 8 | 0.03148 |
| GgaAffx.11757.1.S1_at | *XPO7* | 73 | 3 | 0.03175 |
| GgaAffx.22623.1.S1_at | *ZBED4* | 76 | 6 | 0.03178 |
| Gga.7758.1.S1_at | *TCERG1L* | 48 | 5 | 0.03214 |
| Gga.19361.1.S1_s_at | *TNRC6C* | 75 | 6 | 0.03234 |
| GgaAffx.7736.1.S1_at | *RAB3GAP1* | 77 | 2 | 0.03243 |
| Gga.5999.3.S1_s_at | *PSME3* | 87 | 6 | 0.03256 |
| Gga.8157.1.S1_at | *SNRPD1* | 37 | 6 | 0.03269 |
| Gga.15961.1.S1_s_at | *CEP76* | 76 | 7 | 0.03284 |
| GgaAffx.11397.1.S1_at | *MRPS7* | 36 | 8 | 0.03299 |
| GgaAffx.7458.1.S1_at | *CCDC14* | 77 | 2 | 0.03353 |
| GgaAffx.12914.1.S1_at | *SLC30A6* | 26 | 7 | 0.03373 |
| Gga.12030.1.S1_a_at | *PEX16* | 46 | 4 | 0.03389 |
| Gga.1029.1.S1_a_at | *RBBP4* | 45 | 4 | 0.03396 |
| GgaAffx.9310.1.S1_s_at | *SLC12A2* | 3 | 1 | 0.034 |
| GgaAffx.21329.1.S1_s_at | *DNAJB14* | 22 | 7 | 0.03419 |
| GgaAffx.23065.1.S1_s_at | *USP38* | 75 | 4 | 0.03435 |
| GgaAffx.12419.1.S1_s_at | *DTL* | 77 | 3 | 0.03437 |
| Gga.6315.1.S1_s_at | *KCTD2* | 14 | 4 | 0.03445 |
| Gga.4694.1.S1_s_at | *CCDC72* | 55 | 3 | 0.03447 |
| Gga.987.1.S1_at | *TOMM20* | 74 | 5 | 0.03451 |
| GgaAffx.11539.1.S1_s_at | *SMU1* | 77 | 5 | 0.03452 |
| GgaAffx.11017.5.S1_s_at | *DYNC2H1* | 86 | 4 | 0.03473 |
| Gga.3179.2.S1_at | *TUBA1C* | 50 | 7 | 0.03541 |
| Gga.11276.1.S1_at | *ATP5L* | 32 | 3 | 0.03551 |
| GgaAffx.11521.1.S1_at | *KDM4B* | 56 | 1 | 0.03572 |
| GgaAffx.22475.1.S1_s_at | *COPG2* | 76 | 5 | 0.03578 |
| Gga.4108.4.S1_x_at | *TPM1* | 76 | 6 | 0.03583 |
| Gga.13970.1.S1_s_at | *RBM46* | 60 | 7 | 0.03622 |
| Gga.16542.1.S1_at | *TMEM106B* | 47 | 6 | 0.03654 |
| GgaAffx.4520.1.S1_s_at | *KIF5B* | 81 | 6 | 0.03656 |
| GgaAffx.22120.1.S1_s_at | *POLR3E* | 76 | 3 | 0.03671 |
| Gga.16559.1.S1_at | *SUCLG1* | 21 | 4 | 0.03678 |
| Gga.3379.1.S1_at | *SH3KBP1* | 49 | 5 | 0.03701 |
| Gga.685.1.S1_at | *RAF1* | 81 | 4 | 0.03704 |
| GgaAffx.25374.1.S1_at | *WDR33* | 2 | 1 | 0.03706 |
| GgaAffx.12819.1.S1_at | *TIMM9* | 86 | 4 | 0.03729 |
| Gga.5426.2.S1_s_at | *CD151* | 9 | 2 | 0.03768 |
| GgaAffx.2307.1.S1_s_at | *NUP155* | 73 | 2 | 0.03782 |
| GgaAffx.23188.1.A1_s_at | *NPM3* | 45 | 5 | 0.03791 |
| GgaAffx.12958.1.S1_at | *EVL* | 40 | 6 | 0.03845 |
| GgaAffx.2105.1.S1_s_at | *HERC1* | 76 | 6 | 0.03877 |
| Gga.5518.1.S1_at | *NDUFC1* | 29 | 6 | 0.03902 |
| GgaAffx.13133.1.S1_s_at | *BPGM* | 83 | 8 | 0.03913 |
| GgaAffx.24937.1.S1_s_at | *MCPH1* | 86 | 8 | 0.03945 |
| Gga.7015.1.S1_at | *FPGT* | 40 | 2 | 0.03992 |
| Gga.11449.1.S1_at | *MLLT6* | 43 | 7 | 0.03994 |
| Gga.10938.1.S1_at | *SGCG* | 40 | 3 | 0.03998 |
| Gga.1984.2.A1_a_at | *MAD2L1* | 48 | 5 | 0.04004 |
| GgaAffx.5619.1.S1_at | *PIK3CA* | 76 | 3 | 0.04014 |
| GgaAffx.23279.1.S1_s_at | *AHCTF1* | 82 | 5 | 0.04014 |
| GgaAffx.11970.1.S1_at | *NFE2L1* | 58 | 4 | 0.04041 |
| GgaAffx.7936.2.S1_s_at | *BRD9* | 88 | 3 | 0.04079 |
| GgaAffx.23974.1.S1_at | *CDKAL1* | 10 | 4 | 0.04083 |
| Gga.3497.1.S1_at | *FEM1C* | 75 | 8 | 0.04085 |
| Gga.8877.1.S1_at | *ATXN7L1* | 62 | 6 | 0.04094 |
| Gga.15808.1.S1_at | *CHTOP* | 50 | 2 | 0.04097 |
| Gga.4565.3.S1_a_at | *TEX264* | 12 | 2 | 0.04127 |
| GgaAffx.9617.1.S1_at | *GPR15* | 10 | 1 | 0.04148 |
| GgaAffx.13128.1.S1_at | *SH3BP2* | 81 | 1 | 0.04182 |
| GgaAffx.972.1.S1_at | *NCAPD3* | 80 | 5 | 0.04208 |
| Gga.2170.1.S1_at | *GRB2* | 51 | 8 | 0.04218 |
| Gga.3917.2.S1_a_at | *RPLP1* | 38 | 4 | 0.04229 |
| GgaAffx.20955.1.S1_at | *IPO7* | 56 | 3 | 0.04247 |
| Gga.5435.1.S1_at | *RRAGB* | 34 | 7 | 0.0425 |
| GgaAffx.10163.1.S1_s_at | *RIMS1* | 75 | 5 | 0.04265 |
| Gga.9188.1.S1_a_at | *ATP5G3* | 50 | 2 | 0.0428 |
| GgaAffx.12718.1.S1_s_at | *ATP6V0C* | 32 | 2 | 0.04309 |
| GgaAffx.7567.1.S1_at | *MNAT1* | 4 | 1 | 0.04322 |
| Gga.12880.2.S1_s_at | *SEMA6D* | 22 | 6 | 0.04324 |
| Gga.11708.1.S1_at | *SHPK* | 41 | 8 | 0.04328 |
| Gga.6961.1.S1_at | *TMEM50A* | 87 | 6 | 0.04354 |
| GgaAffx.23445.1.S1_s_at | *PTPN13* | 75 | 6 | 0.04362 |
| Gga.5911.2.S1_a_at | *CLCN3* | 74 | 3 | 0.04423 |
| Gga.16396.1.S1_s_at | *FAF2* | 13 | 4 | 0.04454 |
| Gga.19229.1.S1_at | *LOC427001* | 66 | 5 | 0.04459 |
| GgaAffx.24365.1.A1_s_at | *NUDCD3* | 11 | 3 | 0.04476 |
| GgaAffx.24823.1.S1_s_at | *SEC14L1* | 82 | 4 | 0.04519 |
| GgaAffx.20808.1.S1_at | *TCTN1* | 16 | 3 | 0.04538 |
| Gga.16393.1.S1_at | *ACAD8* | 21 | 4 | 0.04539 |
| Gga.5727.2.S1_a_at | *RG9MTD1* | 14 | 3 | 0.04579 |
| Gga.7108.2.S1_a_at | *TMEM14A* | 13 | 3 | 0.04587 |
| GgaAffx.3491.1.S1_at | *MSI2* | 60 | 5 | 0.04591 |
| Gga.19105.1.S1_s_at | *CTNND1* | 45 | 4 | 0.04596 |
| GgaAffx.8765.2.S1_at | *GFPT2* | 80 | 6 | 0.04602 |
| Gga.19738.1.S1_s_at | *DIAPH3* | 76 | 8 | 0.04605 |
| GgaAffx.9641.1.S1_at | *PCSK5* | 75 | 4 | 0.04611 |
| Gga.11747.1.S1_at | *MTIF3* | 55 | 3 | 0.04625 |
| Gga.4894.1.S1_s_at | *NACA* | 47 | 5 | 0.04646 |
| Gga.5208.2.S1_x_at | *UBQLN1* | 68 | 6 | 0.04654 |
| Gga.12147.1.S1_at | *LYRM1* | 80 | 3 | 0.04658 |
| Gga.16806.3.S1_at | *HOMER3* | 76 | 3 | 0.04672 |
| Gga.13541.1.S1_s_at | *FAR1* | 77 | 7 | 0.0469 |
| Gga.10191.1.S1_at | *WBP4* | 73 | 6 | 0.04691 |
| Gga.14486.1.S1_at | *CASP2* | 4 | 2 | 0.04697 |
| GgaAffx.11662.1.S1_s_at | *OLA1* | 30 | 8 | 0.04712 |
| GgaAffx.22781.1.S1_at | *SESTD1* | 80 | 2 | 0.04723 |
| GgaAffx.2818.1.S1_at | *DPY19L3* | 76 | 2 | 0.04742 |
| Gga.10748.1.S1_at | *MPZL2* | 12 | 3 | 0.04761 |
| Gga.17048.1.S1_at | *MFSD1* | 40 | 7 | 0.04775 |
| Gga.19285.1.S1_at | *SLC22A5* | 28 | 5 | 0.04781 |
| Gga.13173.1.S1_at | *PTER* | 14 | 3 | 0.04856 |
| Gga.3690.1.A1_at | *ZNF207* | 2 | 1 | 0.04859 |
| GgaAffx.3956.1.S1_s_at | *PAXIP1* | 76 | 7 | 0.04891 |
| Gga.18837.1.S1_at | *SMC6* | 36 | 8 | 0.04912 |
| Gga.10064.1.S1_at | *ADAT2* | 74 | 7 | 0.04913 |
| GgaAffx.20932.1.S1_s_at | *ZNF511* | 42 | 4 | 0.04936 |
| Gga.17237.1.S1_at | *STAU1* | 43 | 2 | 0.04959 |
| Gga.1086.1.S1_a_at | *PTRH2* | 53 | 5 | 0.04969 |
| GgaAffx.12961.1.S1_at | *TPX2* | 50 | 5 | 0.04979 |
