# Supplementary material for: Comparison between Timelines of Transcriptional Regulation in Mammals, Birds, and Teleost Fish Somitogenesis
Source: PLoS One. 2016 May 18;11(5):e0155802. doi: 10.1371/journal.pone.0155802 (PMC4871587; doi:10.1371/journal.pone.0155802)
Supplement: S2 Table — The timing of genes found with two peaks of expression during chicken somitogenesis, ranked according to their LS p-value and the regularity of the profile. Times in minutes assume a 90mn periodicity for every transcript and errors are computed by adding to the original transcript source of noise typically found in microarray experiments. (DOCX) [file pone.0155802.s004.docx]

**S2 Table: The list of genes with two peaks of expression during chicken somitogenesis**.

| **Probe Set ID** | **Gene** | **T1(min)** | **err1(min)** | **T2(min)** | **err2(min)** | **LS p-value** |
| --- | --- | --- | --- | --- | --- | --- |
| GgaAffx.7727.2.S1_s_at | *BBS9* | 6 | 1 | 56 | 2 | 0.0024 |
| GgaAffx.12279.1.S1_s_at | *SUCLA2* | 8 | 1 | 53 | 1 | 0.0041 |
| Gga.15350.1.S1_at | *CEP55* | 30 | 2 | 76 | 2 | 0.0042 |
| GgaAffx.4444.2.S1_s_at | *MLEC* | 13 | 1 | 56 | 2 | 0.0056 |
| Gga.16578.1.S1_at | *ORAOV1* | 23 | 1 | 65 | 2 | 0.007 |
| GgaAffx.23567.1.S1_at | *ARPC2* | 14 | 4 | 60 | 2 | 0.011 |
| Gga.13464.1.S1_at | *XAB1* | 12 | 3 | 56 | 3 | 0.0111 |
| GgaAffx.24722.1.S1_s_at | *MAP3K7* | 9 | 2 | 58 | 3 | 0.0113 |
| GgaAffx.13136.1.S1_at | *RAB33B* | 35 | 3 | 83 | 4 | 0.012 |
| Gga.14412.1.S1_at | *C4H4orf32* | 22 | 2 | 72 | 3 | 0.0123 |
| Gga.685.1.S2_at | RAF1 | 12 | 2 | 78 | 4 | 0.0125 |
| Gga.4916.1.S2_s_at | *PDCD4* | 38 | 4 | 85 | 5 | 0.0142 |
| GgaAffx.20873.1.S1_at | *PRKRIR* | 40 | 2 | 86 | 1 | 0.0142 |
| Gga.4598.1.S1_a_at | *PFDN1* | 14 | 1 | 58 | 2 | 0.0143 |
| Gga.5715.1.S1_at | *IFT52* | 14 | 2 | 58 | 3 | 0.0166 |
| Gga.5236.2.S1_a_at | *CWC15* | 13 | 3 | 58 | 3 | 0.017 |
| Gga.1842.1.S1_at | *LLPH* | 16 | 4 | 63 | 4 | 0.0171 |
| Gga.2992.3.S1_x_at | *AKR1A1* | 15 | 1 | 61 | 2 | 0.0171 |
| Gga.5690.1.S1_s_at | *RBM38* | 26 | 1 | 75 | 2 | 0.0192 |
| Gga.5623.2.S1_s_at | *IDH3B* | 14 | 1 | 67 | 3 | 0.0197 |
| Gga.4893.1.S1_at | *AIP* | 18 | 4 | 59 | 4 | 0.0202 |
| Gga.5698.2.S1_a_at | *GMDS* | 20 | 5 | 68 | 6 | 0.0213 |
| GgaAffx.12699.1.S1_s_at | *RANBP1* | 11 | 2 | 58 | 3 | 0.0227 |
| Gga.18096.1.S1_s_at | *WBSCR22* | 14 | 3 | 56 | 3 | 0.0234 |
| Gga.10819.2.S1_s_at | *FBXO30* | 15 | 5 | 58 | 2 | 0.024 |
| Gga.15389.1.S1_s_at | *TRMT12* | 5 | 1 | 52 | 2 | 0.0242 |
| Gga.5931.1.S1_s_at | *BCAS2* | 10 | 2 | 55 | 2 | 0.0244 |
| Gga.18696.1.S1_s_at | *TMCO3* | 15 | 1 | 66 | 4 | 0.027 |
| Gga.8338.1.S1_at | *NIP7* | 13 | 2 | 54 | 3 | 0.029 |
| Gga.7319.2.S1_a_at | *MEF2BNB* | 20 | 5 | 63 | 3 | 0.0291 |
| GgaAffx.6866.1.S1_at | *TWISTNB* | 25 | 6 | 65 | 3 | 0.0296 |
| Gga.19818.1.S1_s_at | *PLEKHO2* | 6 | 1 | 53 | 1 | 0.0304 |
| Gga.9600.1.S1_at | *DCTN5* | 21 | 4 | 68 | 4 | 0.0308 |
| GgaAffx.20612.1.S1_s_at | *C7H2orf69* | 24 | 3 | 75 | 4 | 0.031 |
| Gga.11554.1.S1_at | *ALG2* | 17 | 1 | 63 | 1 | 0.0312 |
| GgaAffx.7971.2.S1_s_at | *PKP4* | 14 | 3 | 59 | 3 | 0.0314 |
| Gga.9081.1.S1_at | *DNAJC3* | 13 | 2 | 55 | 2 | 0.0325 |
| Gga.4712.2.S1_at | *OXCT1* | 22 | 2 | 70 | 3 | 0.0331 |
| Gga.8657.1.S1_at | *PKN2* | 34 | 9 | 76 | 3 | 0.0333 |
| GgaAffx.11527.1.S1_at | *DDT* | 16 | 1 | 60 | 2 | 0.0343 |
| Gga.12868.1.S1_at | *BCCIP* | 15 | 3 | 58 | 3 | 0.0349 |
| Gga.8304.1.S1_at | *DNAJC27* | 10 | 3 | 55 | 4 | 0.0355 |
| Gga.4643.1.S1_at | *ESD* | 12 | 3 | 58 | 3 | 0.0355 |
| Gga.16526.1.S1_at | *ARL9* | 24 | 3 | 73 | 4 | 0.0364 |
| GgaAffx.7070.1.S1_s_at | *CCNK* | 11 | 2 | 58 | 4 | 0.0365 |
| GgaAffx.9790.1.S1_s_at | *HJURP* | 30 | 6 | 79 | 6 | 0.0365 |
| Gga.19902.1.S1_at | *FAF1* | 28 | 4 | 74 | 2 | 0.0368 |
| GgaAffx.20085.1.S1_s_at | *SOX5* | 5 | 1 | 55 | 1 | 0.0373 |
| Gga.18988.1.S1_at | *MADD* | 30 | 5 | 76 | 3 | 0.0381 |
| Gga.2027.1.A1_at | *POP5* | 4 | 1 | 57 | 2 | 0.0388 |
| GgaAffx.5352.3.S1_s_at | *VPS18* | 22 | 1 | 65 | 2 | 0.0391 |
| Gga.2668.1.S1_a_at | *RARB* | 13 | 2 | 60 | 3 | 0.0397 |
| GgaAffx.22491.4.S1_s_at | *SPTBN1* | 4 | 1 | 53 | 1 | 0.0401 |
| GgaAffx.12162.1.S1_at | *PPHLN1* | 42 | 15 | 1 | 1 | 0.0412 |
| GgaAffx.23667.1.S1_at | *HIST1H1C* | 10 | 2 | 53 | 1 | 0.0419 |
| Gga.3363.3.S1_a_at | *IRF2* | 4 | 1 | 57 | 2 | 0.042 |
| GgaAffx.1567.1.S1_at | *OAZ2* | 14 | 3 | 63 | 4 | 0.042 |
| Gga.10731.1.S1_at | *C3H1orf31* | 11 | 3 | 58 | 3 | 0.0426 |
| GgaAffx.11842.1.S1_s_at | *RAP2C* | 22 | 3 | 66 | 3 | 0.0428 |
| GgaAffx.5908.1.S1_s_at | *PRKCI* | 25 | 4 | 72 | 4 | 0.0429 |
| GgaAffx.21081.1.S1_s_at | *ZNF821* | 23 | 3 | 63 | 3 | 0.0429 |
| Gga.5058.1.S1_s_at | *CST3* | 12 | 2 | 58 | 4 | 0.0431 |
| Gga.19869.1.S1_s_at | *TOE1* | 4 | 1 | 58 | 4 | 0.0432 |
| GgaAffx.24191.1.S1_s_at | *PWWP2A* | 12 | 2 | 62 | 5 | 0.0434 |
| Gga.12260.1.S1_at | *IRAK1BP1* | 14 | 4 | 57 | 3 | 0.0434 |
| Gga.11991.1.S1_at | *POLR2C* | 14 | 2 | 58 | 3 | 0.0435 |
| Gga.10782.1.S1_at | *FADD* | 14 | 2 | 54 | 2 | 0.0436 |
| GgaAffx.12476.1.S1_s_at | *RNF126* | 35 | 1 | 86 | 3 | 0.0442 |
| Gga.306.1.S1_at | *WNT11* | 10 | 2 | 57 | 2 | 0.0442 |
| GgaAffx.25149.1.S1_at | *TRIM13* | 16 | 2 | 70 | 3 | 0.0443 |
| GgaAffx.6614.1.S1_at | *SAMD15* | 15 | 5 | 58 | 2 | 0.0444 |
| Gga.11697.1.S1_at | *PRADC1* | 14 | 2 | 59 | 3 | 0.0445 |
| GgaAffx.9018.1.S1_at | *NUP50* | 13 | 2 | 58 | 2 | 0.0449 |
| Gga.2537.2.S1_at | *HMGCL* | 12 | 3 | 57 | 2 | 0.0454 |
| GgaAffx.11032.1.S1_at | *ARHGAP42* | 22 | 1 | 68 | 2 | 0.0454 |
| GgaAffx.4717.1.S1_at | *LOC420491* | 20 | 1 | 68 | 3 | 0.0456 |
| Gga.17824.1.S1_s_at | *RAB5B* | 22 | 2 | 72 | 2 | 0.0463 |
| GgaAffx.20247.1.S1_s_at | *PPFIA1* | 31 | 4 | 76 | 1 | 0.0468 |
| Gga.2283.1.S2_at | *DLL1* | 30 | 6 | 77 | 7 | 0.047 |
| Gga.18623.1.S1_at | *SIRT4* | 17 | 3 | 59 | 2 | 0.047 |
| GgaAffx.728.3.S1_s_at | *ANKHD1* | 48 | 9 | 1 | 1 | 0.0484 |
| Gga.16117.1.A1_at | *CP* | 17 | 2 | 60 | 1 | 0.0486 |
| Gga.12612.1.S1_at | *COX18* | 12 | 2 | 53 | 1 | 0.0488 |
| Gga.3224.1.S1_s_at | *PPP1R8* | 10 | 2 | 51 | 1 | 0.0499 |
