# Supplementary material for: Comparison between Timelines of Transcriptional Regulation in Mammals, Birds, and Teleost Fish Somitogenesis
Source: PLoS One. 2016 May 18;11(5):e0155802. doi: 10.1371/journal.pone.0155802 (PMC4871587; doi:10.1371/journal.pone.0155802)
Supplement: S3 Table — The timing of genes found with one peak of expression during zebrafish somitogenesis, ranked according to their LS p-value and the regularity of the profile. Times in minutes assume a 30mn periodicity for every transcript and errors are computed by adding to the original transcript source of noise typically found in microarray experiments. (DOCX) [file pone.0155802.s005.docx]

**S3 Table: The list of genes with one peak of expression during zebrafish somitogenesis**.

| **Probe set ID** | **Gene** | **Time(min)** | **Err(min)** | **LS p-value** |
| --- | --- | --- | --- | --- |
| Dr.8086.1.S1_s_at | *dlc* | 17 | 1 | 0.0009 |
| Dr.5372.1.S1_x_at | *her4.2* | 25 | 1 | 0.0014 |
| Dr.3696.1.S1_at | *her7* | 24 | 1 | 0.0023 |
| Dr.24815.2.S1_at | *rps29* | 14 | 4 | 0.0024 |
| Dr.11157.1.S1_at | *LOC100003640* | 25 | 2 | 0.0039 |
| Dr.1462.1.S1_at | *her1* | 16 | 1 | 0.0044 |
| Dr.5759.1.A1_at | *hoxd11a* | 6 | 2 | 0.0053 |
| Dr.1899.1.S1_at | *her15.1* | 24 | 1 | 0.0058 |
| Dr.4733.1.A1_at | *spty2d1* | 2 | 2 | 0.0063 |
| Dr.7852.1.S1_at | *utp11l* | 28 | 4 | 0.0072 |
| Dr.8835.2.S1_at | *mphosph8* | 13 | 4 | 0.008 |
| Dr.1410.1.S1_at | *prrx1a* | 29 | 5 | 0.0083 |
| Dr.4295.1.S1_at | *rfc4* | 10 | 3 | 0.0087 |
| Dr.17281.1.A1_at | *zgc:152990* | 18 | 4 | 0.0097 |
| Dr.1460.1.S1_at | *her2* | 27 | 2 | 0.0106 |
| Dr.12851.1.S1_at | *mrpl40* | 24 | 5 | 0.0109 |
| Dr.14998.2.S1_at | *ech1* | 28 | 5 | 0.0111 |
| Dr.14781.1.S1_at | *ubxn7* | 14 | 3 | 0.012 |
| Dr.17394.1.A1_at | *lmf2b* | 18 | 5 | 0.0129 |
| Dr.13969.1.A1_at | *nipa2* | 14 | 3 | 0.0129 |
| Dr.25598.1.A1_at | *h2afy2* | 6 | 1 | 0.0146 |
| Dr.5545.1.S1_at | *rpl32* | 16 | 4 | 0.0148 |
| Dr.1055.1.S1_at | *mcm7* | 14 | 3 | 0.0153 |
| Dr.2500.1.S1_at | *hs6st2* | 12 | 4 | 0.0155 |
| Dr.422.1.A1_at | *wu:fb74b10* | 16 | 4 | 0.0165 |
| Dr.20265.1.S1_at | *sptlc3* | 15 | 5 | 0.0175 |
| Dr.4169.1.A1_at | *nars* | 20 | 5 | 0.0177 |
| Dr.4142.1.A1_at | *zgc:158452* | 12 | 2 | 0.0178 |
| Dr.8001.6.S1_a_at | *rps26* | 15 | 5 | 0.0181 |
| Dr.635.1.S1_at | *atp5l* | 16 | 3 | 0.0191 |
| Dr.4831.1.A1_at | *hiat1a* | 12 | 3 | 0.0194 |
| Dr.560.2.S1_at | *fbln1* | 23 | 1 | 0.02 |
| Dr.20340.1.A1_at | *zgc:77235* | 16 | 2 | 0.0205 |
| Dr.3131.1.S1_at | *rps8a* | 15 | 4 | 0.0223 |
| Dr.13042.1.S1_at | *rad23aa* | 18 | 5 | 0.0223 |
| Dr.3183.1.A1_at | *wu:fc28f08* | 29 | 5 | 0.0225 |
| Dr.7626.1.A1_at | *pfkfb3* | 28 | 4 | 0.0229 |
| Dr.5648.1.A1_at | *ewsr1a* | 14 | 3 | 0.0233 |
| Dr.12368.1.A1_at | *atpaf1* | 1 | 1 | 0.0236 |
| Dr.25528.1.A1_at | *btbd6b* | 16 | 1 | 0.0237 |
| Dr.4585.1.S1_at | *pdcd5* | 23 | 5 | 0.0241 |
| Dr.14716.1.A1_at | *tiprl* | 12 | 5 | 0.0246 |
| Dr.25829.1.A1_at | *dmap1* | 17 | 1 | 0.0249 |
| Dr.12056.1.A1_at | *ergic2* | 26 | 5 | 0.025 |
| Dr.19508.1.A1_at | *zgc:77285* | 18 | 5 | 0.0254 |
| Dr.2634.1.A1_at | *enpp1* | 25 | 5 | 0.0262 |
| Dr.3520.1.S1_at | *gmcl1* | 26 | 5 | 0.027 |
| Dr.15025.1.S1_at | *zgc:77086* | 21 | 3 | 0.0273 |
| Dr.8026.1.A1_at | *commd2* | 8 | 1 | 0.0277 |
| Dr.20951.1.S1_at | *sypl2a* | 26 | 5 | 0.0284 |
| Dr.1111.1.S1_at | *ddx39aa* | 9 | 3 | 0.0285 |
| Dr.6660.1.S1_at | *dhx16* | 12 | 2 | 0.0288 |
| Dr.12656.1.S1_at | *mios* | 3 | 1 | 0.029 |
| Dr.705.1.A1_at | *rnps1* | 16 | 5 | 0.029 |
| Dr.18319.1.S1_at | *si:dkeyp-50f7.2* | 29 | 5 | 0.0294 |
| Dr.15443.2.S1_at | *rpl10a* | 16 | 4 | 0.0296 |
| Dr.1338.1.S1_at | *rpl28* | 15 | 5 | 0.0301 |
| Dr.1340.1.S1_at | *uba52* | 15 | 4 | 0.0319 |
| Dr.7508.1.A1_at | *wu:fd08h09* | 19 | 5 | 0.0326 |
| Dr.15867.1.S1_at | *rpl34* | 15 | 5 | 0.0327 |
| Dr.172.1.S1_at | *trpc4apa* | 8 | 2 | 0.0333 |
| Dr.16322.1.A1_at | *add1* | 14 | 2 | 0.0333 |
| Dr.1993.1.S1_at | *cul1a* | 8 | 2 | 0.0339 |
| Dr.4771.1.S1_at | *ndufb9* | 14 | 4 | 0.0352 |
| Dr.3258.1.A1_at | *zgc:158415* | 27 | 5 | 0.0356 |
| Dr.1351.1.S1_at | *rpl17* | 16 | 4 | 0.0358 |
| Dr.4412.4.S1_at | *rps4x* | 15 | 4 | 0.036 |
| Dr.17543.3.A1_at | *zgc:66160* | 27 | 5 | 0.0368 |
| Dr.3155.1.S1_at | *trmt11* | 25 | 5 | 0.0368 |
| Dr.5098.1.S1_at | *rpl35* | 16 | 5 | 0.0369 |
| Dr.3995.1.S1_at | *cltca* | 14 | 5 | 0.0375 |
| Dr.1345.1.S1_at | *rps23* | 16 | 5 | 0.0376 |
| Dr.18762.1.S1_at | *rhoae* | 8 | 2 | 0.0376 |
| Dr.9544.1.S1_at | *ilk* | 3 | 1 | 0.0377 |
| Dr.24320.1.S1_at | *bnip4* | 27 | 5 | 0.0379 |
| Dr.23832.1.S1_at | *rfx1b* | 1 | 1 | 0.0387 |
| Dr.184.1.S1_at | *b2m* | 16 | 3 | 0.0393 |
| Dr.1282.2.S1_at | *rps26l* | 17 | 5 | 0.0401 |
| Dr.25159.1.S1_at | *bckdk* | 29 | 5 | 0.0403 |
| Dr.25912.1.A1_at | *ghra* | 18 | 1 | 0.0404 |
| Dr.16557.1.S1_at | *wu:ft63e05* | 13 | 2 | 0.0409 |
| Dr.786.1.S1_at | *psma2* | 8 | 2 | 0.041 |
| Dr.16499.1.A1_at | *nr3c1* | 23 | 5 | 0.041 |
| Dr.16880.1.S1_at | *pcdh18a* | 3 | 1 | 0.041 |
| Dr.1310.2.S1_a_at | *rpl24* | 15 | 4 | 0.0413 |
| Dr.1618.1.S1_at | *kiaa0907* | 14 | 4 | 0.0421 |
| Dr.3514.1.S1_at | *emc8* | 28 | 2 | 0.0423 |
| Dr.10309.1.S1_at | *im:6906574* | 12 | 1 | 0.0427 |
| Dr.8155.1.S1_at | *med17* | 11 | 3 | 0.0432 |
| Dr.280.2.S1_at | *rps20* | 15 | 3 | 0.0432 |
| Dr.26334.1.A1_at | *srpk1a* | 29 | 5 | 0.0434 |
| Dr.11560.1.A1_at | *si:dkeyp-110e4.6* | 21 | 5 | 0.0436 |
| Dr.2277.1.A1_at | *eps8l1* | 2 | 1 | 0.0438 |
| Dr.743.1.S1_at | *pomp* | 16 | 5 | 0.0449 |
| Dr.2478.1.S1_at | *erp44* | 7 | 1 | 0.0454 |
| Dr.9746.3.S1_at | *rpl19* | 15 | 4 | 0.0455 |
| Dr.41.1.A1_at | *msxe* | 14 | 5 | 0.0458 |
| Dr.14394.1.S1_at | *tceb1a* | 29 | 5 | 0.0465 |
| Dr.11713.1.S1_at | *uprt* | 2 | 2 | 0.0466 |
| Dr.5969.1.S1_at | *use1* | 26 | 5 | 0.0467 |
| Dr.26462.1.S1_at | *zdhhc16b* | 12 | 4 | 0.0468 |
| Dr.15090.1.A1_at | *ints10* | 15 | 3 | 0.0469 |
| Dr.18150.1.S1_at | *exosc9* | 22 | 5 | 0.0469 |
| Dr.10742.2.S1_a_at | *hspbp1* | 10 | 1 | 0.0471 |
| Dr.5991.1.A1_at | *nsun4* | 2 | 1 | 0.0473 |
| Dr.959.2.S1_at | *btf3* | 12 | 1 | 0.0474 |
| Dr.10141.1.A1_at | *angptl7* | 25 | 5 | 0.0481 |
| Dr.245.1.A1_at | *zgc:92172* | 15 | 4 | 0.0484 |
| Dr.15127.1.S1_at | *kbtbd4* | 11 | 3 | 0.0485 |
| Dr.24498.1.A1_at | *tmem169* | 28 | 5 | 0.0486 |
| Dr.953.2.S1_at | *rps16* | 14 | 4 | 0.0498 |
