# Supplementary material for: Comparison between Timelines of Transcriptional Regulation in Mammals, Birds, and Teleost Fish Somitogenesis
Source: PLoS One. 2016 May 18;11(5):e0155802. doi: 10.1371/journal.pone.0155802 (PMC4871587; doi:10.1371/journal.pone.0155802)
Supplement: S4 Table — The timing of genes found with two peaks of expression during zebrafish somitogenesis, ranked according to their LS p-value and the regularity of the profile. Times in minutes assume a 30mn periodicity for every transcript and errors are computed by adding to the original transcript source of noise typically found in microarray experiments. (DOCX) [file pone.0155802.s006.docx]

**S4 Table: The list of genes with two peaks of expression during zebrafish somitogenesis.**

| **Probe set ID** | **Gene** | **T1(min)** | **err1(min)** | **T2(min)** | **err2(min)** | **LS p-value** |
| --- | --- | --- | --- | --- | --- | --- |
| Dr.23663.1.S1_at | *atn1* | 2 | 2 | 15 | 3 | 0.0092 |
| Dr.14917.1.A1_at | *ccdc22* | 7 | 1 | 22 | 1 | 0.0106 |
| Dr.20000.1.S1_at | *tfam* | 10 | 1 | 25 | 1 | 0.0117 |
| Dr.15883.1.S1_at | *ppp2r5eb* | 8 | 2 | 22 | 1 | 0.013 |
| Dr.24399.1.A1_at | *arglu1b* | 8 | 1 | 23 | 1 | 0.0138 |
| Dr.4392.1.A1_at | *lzts2a* | 2 | 1 | 15 | 1 | 0.0141 |
| Dr.25185.2.S1_x_at | *si:busm1-79m10.1* | 1 | 2 | 16 | 1 | 0.0162 |
| Dr.23451.1.S1_s_at | *fzd8a* | 9 | 1 | 25 | 1 | 0.0173 |
| Dr.4651.1.S1_at | *zgc:92744* | 6 | 1 | 21 | 1 | 0.0191 |
| Dr.9964.2.S1_at | *akap9* | 8 | 2 | 22 | 1 | 0.0195 |
| Dr.13182.1.A1_at | *rab5b* | 6 | 1 | 21 | 1 | 0.0217 |
| Dr.3076.2.S1_at | *kif15* | 8 | 1 | 23 | 1 | 0.0235 |
| Dr.25444.1.A1_at | *im:7136583* | 6 | 1 | 21 | 2 | 0.0239 |
| Dr.20334.1.S1_at | *zgc:101000* | 4 | 1 | 18 | 3 | 0.025 |
| Dr.14379.1.S1_at | *si:dkeyp-94h10.5* | 2 | 2 | 16 | 5 | 0.0283 |
| Dr.5122.1.S2_at | *hnrnpa0a* | 1 | 2 | 14 | 1 | 0.0284 |
| Dr.24314.1.S1_at | *rapgef1b* | 8 | 1 | 23 | 2 | 0.0286 |
| Dr.18540.2.S1_a_at | *si:busm1-48c11.1* | 10 | 1 | 24 | 4 | 0.029 |
| Dr.5793.1.S1_at | *crnkl1* | 5 | 1 | 22 | 1 | 0.0302 |
| Dr.12686.1.A1_at | *atg5* | 9 | 1 | 25 | 1 | 0.0309 |
| Dr.2575.1.A1_at | *zgc:163098* | 9 | 1 | 24 | 1 | 0.0347 |
| Dr.15098.1.A1_at | *slc26a11* | 11 | 3 | 27 | 4 | 0.0354 |
| Dr.16990.1.S1_at | *zgc:86715* | 3 | 1 | 21 | 2 | 0.0362 |
| Dr.14413.2.S1_at | *zgc:77724* | 10 | 1 | 26 | 2 | 0.0365 |
| Dr.24121.1.A1_at | *uggt1* | 10 | 1 | 26 | 2 | 0.0372 |
| Dr.20010.6.A1_at | *mccc2* | 8 | 1 | 21 | 1 | 0.0399 |
| Dr.22984.1.A1_at | *wu:fj82b05* | 4 | 2 | 20 | 4 | 0.0416 |
| Dr.21927.1.A1_at | *wu:fc83f07* | 13 | 2 | 29 | 2 | 0.0441 |
| Dr.14832.1.A1_at | *erbb2ip* | 2 | 1 | 15 | 3 | 0.0447 |
| Dr.2739.1.A1_x_at | *wu:fc59h03* | 2 | 1 | 16 | 5 | 0.0474 |
| Dr.604.1.S2_at | *notch2* | 8 | 4 | 23 | 1 | 0.0481 |
| Dr.24333.1.A1_at | *wu:fa14e09* | 6 | 1 | 20 | 1 | 0.0494 |
