# Supplementary material for: Comparison between Timelines of Transcriptional Regulation in Mammals, Birds, and Teleost Fish Somitogenesis
Source: PLoS One. 2016 May 18;11(5):e0155802. doi: 10.1371/journal.pone.0155802 (PMC4871587; doi:10.1371/journal.pone.0155802)
Supplement: S5 Table — From the set of 1134 genes identified to periodically expressed during cell cycle, only 112 were found to have the GC-rich motif A1 in their promoters. This low overlap (~10%) demonstrates that the GC-rich motif is not significant in the cell cycle, in agreement with previous results in that the cell cycle and the periodic activation of gene expression during somitogenesis are two separate and independent processes. (DOCX) [file pone.0155802.s007.docx]

**S5 Table: The list of known cell cycle genes enriched with the GC-motif A1 in their promoters.**

| **Gene Symbol** | **E-value** | **Gene Symbol** | **E-value** | **Gene Symbol** | **E-value** |
| --- | --- | --- | --- | --- | --- |
| *LMNA* | 1.2e-08 | *TRIP13* | 0.00012 | *POLD3* | 0.003 |
| *CCNE1* | 1.5e-07 | *CASP3* | 0.00012 | *MCM4* | 0.003 |
| *CDH24* | 3.6e-07 | *PDGFA* | 0.00013 | *ITGB3* | 0.003 |
| *SEPN1* | 4.1e-07 | *PPP1R2* | 0.00013 | *BARD1* | 0.003 |
| *INSR* | 5.4e-07 | *CSH2* | 0.00016 | *ANLN* | 0.0032 |
| *BMI1* | 7e-07 | *ADCY6* | 0.00024 | *GNB1* | 0.0035 |
| *ABCC1* | 8e-07 | *PCNA* | 0.00027 | *UACA* | 0.0035 |
| *NFIC* | 8e-07 | *TACC3* | 0.00029 | *EBI3* | 0.0046 |
| *RAD54L* | 9.1e-07 | *HIF1A* | 0.00029 | *RFC2* | 0.005 |
| *B4GALT1* | 9.1e-07 | *PPP2CA* | 0.00029 | *RNPS1* | 0.0059 |
| *MNT* | 9.1e-07 | *E2F1* | 0.00036 | *ACYP1* | 0.0064 |
| *VEGFC* | 1e-06 | *MUC1* | 0.00043 | *CKS2* | 0.0069 |
| *BRD7* | 1e-06 | *CDC25C* | 0.00043 | *CDR2* | 0.0082 |
| *RUNX1* | 1.2e-06 | *BAG3* | 0.00043 | *TIMP1* | 0.01 |
| *LRRFIP1* | 1.5e-06 | *STAG3* | 0.00048 | *CD24* | 0.01 |
| *MLLT4* | 2e-06 | *PTPN9* | 0.00048 | *KIAA1586* | 0.01 |
| *CDC25B* | 2.2e-06 | *MGAT2* | 0.00048 | *NUDT4* | 0.01 |
| *CCND1* | 3.2e-06 | *CDC20* | 0.00058 | *RAN* | 0.011 |
| *CDKN2C* | 6.7e-06 | *ROCK1* | 0.00064 | *AP3D1* | 0.012 |
| *GAS6* | 7.5e-06 | *SLC22A3* | 0.00064 | *ITPR1* | 0.012 |
| *INSM1* | 9.5e-06 | *CTCF* | 0.00077 | *MCM6* | 0.013 |
| *PLAG1* | 1.2e-05 | *MBD4* | 0.00084 | *MKI67* | 0.013 |
| *BAIAP2* | 1.7e-05 | *RECQL4* | 0.00084 | *HRAS* | 0.016 |
| *RERE* | 1.7e-05 | *PKNOX1* | 0.00084 | *PSEN1* | 0.017 |
| *MAN1A2* | 1.9e-05 | *GCLM* | 0.00093 | *EIF4E* | 0.021 |
| *BMP2* | 2.1e-05 | *DSP* | 0.001 | *COL7A1* | 0.025 |
| *ARHGAP8* | 2.4e-05 | *MET* | 0.001 | *RRM2* | 0.027 |
| *CCNB2* | 2.4e-05 | *TXNRD1* | 0.001 | *BRCA1* | 0.029 |
| *GADD45A* | 3e-05 | *MDM2* | 0.001 | *FZR1* | 0.029 |
| *INADL* | 3e-05 | *TSN* | 0.001 | *BIRC2* | 0.031 |
| *H1F0* | 3e-05 | *CAPN7* | 0.0011 | *LMO4* | 0.031 |
| *GAS1* | 3e-05 | *UBQLN2* | 0.0011 | *KPNA2* | 0.031 |
| *UBE2D3* | 6.3e-05 | *DONSON* | 0.0015 | *PRPSAP1* | 0.034 |
| *YWHAH* | 7.1e-05 | *EIF4EBP2* | 0.0016 | *RANGAP1* | 0.039 |
| *DUSP4* | 0.00011 | *CCNF* | 0.0021 | *CDC42* | 0.039 |
| *EZH2* | 0.0023 | *CTSD* | 0.0025 | *FYN* | 0.042 |
| *SRD5A1* | 0.0023 | *PIK3CD* | 0.0027 | *DHFR* | 0.045 |
|  |  |  |  | *CHAF1B* | 0.049 |
