# Supplementary material for: Comparison between Timelines of Transcriptional Regulation in Mammals, Birds, and Teleost Fish Somitogenesis
Source: PLoS One. 2016 May 18;11(5):e0155802. doi: 10.1371/journal.pone.0155802 (PMC4871587; doi:10.1371/journal.pone.0155802)
Supplement: S6 Table — We compare the statistics of the motif analysis for promoter lengths of 1kb, 2kb, 5kb and 10 kb. The 2kb analysis appears to be the best compromise between false positives (high for longer sequences) and false negative (high for too short sequences) and provide better gene coverage. (DOCX) [file pone.0155802.s008.docx]

**S6 Table: Influence of the length of the promoter on the motif analysis**

|  | **1kb** | **2kb** | **5 kb** | **10 kb** |
| --- | --- | --- | --- | --- |
| **Number of motifs** | 10 | 10 | 15 | 25 |
| **Average motif width** | 26 | 27 | 35 | 38 |
| **Gene coverage** | 61.2% | 92.3% | 62.4% | 69.8% |
| **Average E-value** | < 10-30 | < 10-40 | < 10-20 | < 10-30 |
